# Bardet-Biedl syndrome 1 mutations differentially impact BBSome integrity and ciliary trafficking

**DOI:** 10.1186/s12964-026-03006-8

**Published:** 2026-06-19

**Authors:** Kristyna Maskova, Hana Hajsmanova, Sofiia Bykova, Sindija Smite, Avishek Prasai, Daniel Rozbesky, Martina Huranova

**Affiliations:** 1 Laboratory of Cilia Genetics and Pathology, Institute of Molecular Genetics of the Czech Academy of Sciences, Prague, 14220 Czech Republic; 2 Department of Medical Biochemistry and Molecular Biology, Center for Molecular Signaling (PZMS), Saarland University School of Medicine, Homburg, Germany; 3 Laboratory of Structural Neurobiology, Institute of Molecular Genetics of the Czech Academy of Sciences, Prague, 14220 Czech Republic; 4 Department of Cell Biology, Faculty of Science, Charles University, Prague, 12800 Czech Republic

**Keywords:** Bardet-Biedl syndrome, BBSome, BBS1, Cilium, Pericentriolar satellites, GPR161, UbK63, TOM1L2, Intraflagellar transport

## Abstract

**Background:**

Bardet-Biedl syndrome is a pleiotropic ciliopathy marked by retinal degeneration, obesity, polydactyly, renal and reproductive anomalies, and cognitive impairment. *BBS1*, the most frequently mutated gene in Bardet-Biedl syndrome, encodes a key component of the BBSome complex, which is essential for ciliary membrane trafficking. Although BBS1 is known to be essential for proper BBSome function, the effects of disease-associated BBS1 variants on its activity remain incompletely understood.

**Methods:**

In this study, we examined how patient-derived BBS1 mutations affect BBSome integrity and its role in cargo transport within primary cilia.

**Results:**

Our results show that particular BBS1 mutations interfere with distinct stages of BBSome assembly and trafficking. While M390R disrupts initial pre-BBSome assembly at pericentriolar satellites, E224K impairs both the maturation of the pre-BBSome into the BBSome and its movement from pericentriolar satellites to the cilium. In contrast, the R160Q variant preserves BBSome assembly and permits its localization to cilia. It specifically weakens the BBSome-GPCR interaction mediated by TOM1L2, resulting in defective GPR161 export and increased ciliary IFT turnover.

**Conclusions:**

Overall, our study establishes a mechanistic framework linking specific BBS1 mutations to distinct defects in BBSome assembly and function. This framework defines functional classes of BBS1 variants and provides deeper insight into the molecular mechanism and severity of Bardet-Biedl syndrome.

**Supplementary Information:**

The online version contains supplementary material available at 10.1186/s12964-026-03006-8.

## Introduction

Bardet-Biedl syndrome (BBS) (OMIM 209900) is the most common non-lethal multisystemic genetic disorder caused by cilia dysfunction [1, 2]. Primary cilia, hereafter referred to as cilia, are microtubule-based sensory organelles present on the surface of most vertebrate cells and play key roles in cellular signaling [3]. Cilium assembly and maintenance further depend on the centrosome and pericentriolar satellites, which coordinate the trafficking of ciliary proteins to the ciliary compartment [4, 5].

BBS is characterized by a broad spectrum of clinical features, including retinal degeneration, polydactyly, obesity, renal anomalies, cognitive impairment, and hypogonadism [2, 6, 7]. To date, at least 26 genes have been linked to BBS, eight of which encode subunits of the BBSome complex: BBS1, BBS2, BBS4, BBS5, BBS7, BBS8, BBS9, and BBS18 [6, 8, 9]. The BBSome assembles in a stepwise, spatially regulated manner governed by BBS4 and BBS1 in vivo [9, 10]. BBS4 localizes to pericentriolar satellites, where it recruits other subunits to form a pre-BBSome, while BBS1 at the centrosome promotes its translocation and final assembly at the ciliary base [9]. The BBSome then facilitates the export of a subset of G-protein coupled receptors (GPCR) from cilia through interactions with intraflagellar transport (IFT) machinery and TOM1L2, an adapter for ubiquitinated cargo [11–16]. The BBSome regulates multiple signaling pathways, such as Sonic Hedgehog [17–19], WNT [20–22], photoreceptor signaling [23, 24], and GPCR-mediated neuronal signaling [25, 26].

BBS1, a key subunit of the BBSome, is the most commonly mutated gene in BBS, accounting for approximately 28% of cases [6, 27]. Structural and biochemical studies have demonstrated that the BBS1 plays a central role in BBSome assembly and function. It directly interacts with multiple BBSome subunits, contributes to the formation of the cargo-binding sites, binds the small GTPase ARL6/BBS3 to facilitate BBSome activation, membrane recruitment and translocation to cilia [9, 11, 12, 28–31].

BBS1 mutations are typically associated with a milder clinical form of BBS than mutations in some other BBSome encoding genes [6], with the symptom frequency and severity varying depending on the specific mutation involved; substitution mutations also tend to have milder symptoms than larger gene aberrations [6, 32–35]. The most common BBS1 variant is the M390R missense mutation, a major pathogenic allele in BBS [6, 32], likely disrupting the BBS1 stability and interaction with ARL6 and BBS4 [10, 28, 36]. The E224K and R160Q mutations are linked to milder clinical symptoms that, based on clinical presentation alone, may not always fulfil the diagnostic criteria for BBS [2, 7, 33–35, 37]. E224K mutation shows phenotypic overlap with Alström syndrome and is currently classified as likely pathogenic [34, 38]. R160Q is primarily associated with retinal anomalies and affects 5´ splice site, leading to reduced levels of the mutant protein [33, 35]. While additional BBS1 mutations have been identified [38, 39], their pathogenic effects are primarily inferred from structural models of the BBSome [30, 31].

In this study, we found that the BBS1 mutations M390R, E224K, and R160Q can be divided into distinct functional classes: those that disrupt BBSome assembly and its translocation to cilia, and those that permit BBSome assembly but fail to mediate cargo retrieval by connecting the ubiquitinated cargo-TOM1L2 to IFT. These results show unique pathological mechanisms for individual genetic mutations underlying BBS.

## Methods

### Antibodies, dyes and reagents

Mouse anti-acetylated tubulin antibody (in-house clone C3B9 [9]; IF, 1:50) was provided by Dr. Vladimir Varga, Institute of Molecular Genetics of the Czech Academy of Sciences. Mouse anti-FLAG (M2 clone) (F1804; WB, 1:1000), mouse anti-β-Actin (A1978, WB, 1:5000), rabbit anti-BBS9 (HPA021289; IF, 1:250, WB, 1:500), rabbit anti-BBS7 (HPA044592; WB, 1:500) and rabbit anti-UbK63 (05–1308; IF, 1:200) were purchased from Sigma Aldrich. Mouse anti-PCM-1 (sc-398365; IF, 1:50), mouse anti-BBS2 (sc-365355; WB, 1:500) and mouse anti-BBS8 (sc-271009; WB, 1:500) were purchased from Santa Cruz. Rabbit anti-BBS1 (ab166613; WB, 1:500) and rabbit anti-TOM1L2 (ab96320; IF, 1:200) were purchased from Abcam. Rabbit anti-GPR161 (13,398–1-AP; IF, 1:200), rabbit anti-BBS5 (14,569–1-AP; WB, 1:500) and rabbit anti-BBS4 (12,766–1-AP; WB, 1:500) were purchased from Proteintech. Rabbit anti-mCherry (PA534974; IF, 1:100) was purchased from ThermoFisher. Goat anti-GFP (ab6673; IP, 3.2 µg per sample) was purchased from Abcam. Primary conjugated antibody for flow cytometry anti-Thy1.1 PE-Cy7 (25–0900) was purchased from eBioscience.

Secondary antibodies for IF were as follows: anti-mouse Alexa Fluor 647 (Invitrogen, A-21235; 1:1000), anti-rabbit Alexa Fluor 488 (Invitrogen, A11008; 1:1000). Secondary antibodies for WB were as follows: goat anti-rabbit-HRP (Jackson, 111–035–144; 1:10,000), goat anti-mouse-HRP (Jackson, 115–035–146; 1:10,000), bovine anti-goat-HRP (Jackson, 805–035–180; 1:10,000).

SiR-Tubulin (SC002) was purchased from Spirochrome and used at a final concentration of 1 µM. ProLong™ Gold antifade reagent with 4′,6-diamidino-2-phenylindole (DAPI) (P36941) was purchased from Invitrogen.

### Cell cultures and treatments

Immortalized human retinal pigment epithelium cell line, hTERT-RPE-1 (RPE1) (ATCC, CRL-400) was provided by Dr. Vladimir Varga (Institute of Molecular Genetics of the Czech Academy of Sciences). RPE1 *BBS1* KO cell line was established previously [9]. Phoenix Ampho cell line was provided by Dr. Tomas Brdicka (Institute of Molecular Genetics of the Czech Academy of Sciences). All cell lines were cultured in complete Dulbecco’s modified Eagle’s medium (DMEM, Sigma, D6429—500 mL) supplemented with 10% fetal bovine serum (FBS), 100 U/mL penicillin (BB Pharma), 100 μg/mL streptomycin (Sigma-Aldrich), and 40 μg/mL gentamicin (Sandoz). All cell lines are regularly tested for mycoplasma contamination. Smoothened agonist—SAG (566,660; Sigma-Aldrich) was used at a concentration of 200 nM. The treatments were for 2 h if not indicated otherwise.

### Cloning and gene transfections

*BBS1* ORF was amplified from pMSCV-BBS1-YFP [9], appended with mCherry and FLAG coding sequences at the N terminus using recombinant PCR, and cloned pMSCV-IRES-Thy 1.1 vector (Clontech) using BglII and EcoRI restriction sites. PCR based mutagenesis was performed on the mCherry-FLAG-BBS1 open reading frame (ORF), where selected BBS1 mutations were introduced using designed primers: M390R (c.1169 T > G, F: CACCCTCATCAGGACCACTCGA, R: TCGAGTGGTCCTGATGAGGGTG), E224K (c.670G > A, F: TGCTGGGCACCAAGAACAAGGA, R: TCCTTGTTCTTGGTGCCCAGCA) and R160Q (c.479G > A, F: GAGAGCATCCAGGAGACGGCAG, R: CTGCCGTCTCCTGGATGCTCTC).

The viral transduction of cell lines was done according to [40] with slight modifications. For viral particle production, Phoenix Ampho cells were seeded on 10 cm dish and allowed to reach confluency of 60–70%. 30 µg of plasmid DNA was transfected into Phoenix Ampho cells using the polyethyleneimine to generate retroviruses. Cells were incubated overnight, and the media was changed to production media (DMEM + ATB + FBS) on the next day. RPE1 cells were transduced with 2 mL of supernatant containing viral particles along with 8 µg/mL polybrene and analyzed for transgene expression after 48 h. RPE1 cells stably expressing both mCherry and Thy 1.1. markers or YFP were bulk sorted using FACS Aria IIu (BD Biosciences).

### Protein lysates, co-immunoprecipitation and western blotting

RPE1 cells were grown in 15 cm cell culture dishes and lysed in the lysis buffer (20 mM HEPES at pH 7.5, 150 mM NaCl, 2 mM EDTA at pH 8, 0.5% Triton X-100) supplemented with protease inhibitor cocktail (complete, Roche, #05056489001). Lysates were cleared by centrifugation at 15,000 × g for 15 min at 4 °C. Protein concentration was adjusted using the Pierce™ BCA Protein Assay Kit (Thermo Scientific). For co-immunoprecipitation assay, protein lysates were immunoprecipitated with anti-FLAG M2 beads (Sigma Aldrich, A2220) or goat anti-GFP antibody (Abcam, 6673, 3.2 µg per sample) conjugated to Protein A Plus UltraLink beads (ThermoFisher, #53,142) for 1.5 h at 4 °C. Beads were washed three times in lysis buffer and co-precipitated proteins were denatured in 1 × Laemmli buffer. For protein expression analysis, protein lysates were immediately denatured in reducing 4 × Laemmli buffer. Denatured protein samples were analyzed by Western blotting following standard protocols. Membranes were probed with primary antibodies overnight at 4 °C and secondary antibodies for 1 h at room temperature. Membranes were developed using chemiluminescence immunoblot imaging system Fusion Solo S (Vilber).

### Flow cytometry

RPE1 cells expressing the mCherry-FLAG-tagged BBS1 variants were cultured in 6-well dishes, trypsinized and centrifuged at 400 × g for 4 min. The cell pellets were washed in PBS and resuspended in FACS buffer (2 mM EDTA, 2% FBS, and 0.1% sodium azide) containing anti-Thy1.1 PE-Cy7 antibody (eBioscience, 25–0900). Measurements were taken on Cytek Aurora Flow Cytometer (Cytek). Geometric mean fluorescence intensities of mCherry and Thy 1.1 positive cells were obtained and used to examine the protein expression. Flow cytometry data was analyzed in FlowJo software, version 10.x (BD Life Sciences).

### Immunofluorescence

RPE1 cells were seeded on 12 mm coverslips and serum starved for 24 h. After respective treatments, cells were fixed (4% formaldehyde) and permeabilized (0.2% Triton X-100) for 10 min. Optional step after fixation was antigen retrieval (BBS9—Fig. 3D, UbK63 – Fig. 5B, TOM1L2—Fig. 5D), when cells were incubated for 5 min in 95 °C in the pre-heated antigen retrieval buffer (100 mM TRIS, 5% urea, pH 9.5). Blocking was done using 5% goat serum (Sigma, G6767-100 mL) in PBS for 15 min and incubated with primary antibody (1% goat serum/PBS) and secondary antibody (PBS) for 1 h and 45 min, respectively in a wet chamber. The cells were washed after each step in PBS three times. At last, the cells were washed in distilled H_2_O, air-dried, and mounted using ProLong™ Gold antifade reagent with DAPI (P36941; Invitrogen).

### Fluorescence microscopy and data analysis

Image acquisition for BBS9, mCherry and GPR161 localization was performed on the Delta Vision Core microscope using the oil immersion objective (Plan-Apochromat 60 × NA 1.42) and filters for DAPI (435/48), FITC (523/36), TRITC (576/89) and Cy5 (632/22). Z-stacks were acquired at 1024 × 1024-pixel format and Z-steps of 0.2 µm.

Image acquisition for UbK63 and TOM1L2 localization was performed on the Leica DM6000 microscope using the oil immersion objective (HCX PL APO 63 × NA 1.40) and filter cubes for DAPI (LP 425), GFP (525/50), TRITC (600/40) and Cy5 (700/75). Z-stacks were acquired at 2048 × 2048-pixel format and Z-steps of 0.2 µm.

Maximum intensity projections were used to quantify ciliary length based on acetylated tubulin staining (Fig. 3C); frequency of BBS9 localization to cilia (Fig. 3E), BBS9 localization to PS (Fig. 4B), and UbK63 and TOM1L2 localization to cilia (Fig. 5C and E); as well as to measure BBS9 intensity profiles (Fig. 3F) and GPR161 signal intensity (Fig. 2D) within cilia using the Fiji ImageJ. Cilia length was quantified using the line tool. Signal intensity was quantified using the line tool following subtraction of background signal adjacent to the cilium. Intensity distribution along the cilium was analyzed using the Plot Profile function with background subtraction, followed by normalization to the first data point to facilitate comparison between conditions.

### Fluorescence recovery after photobleaching, data processing, and analysis

Cells expressing YFP-BBS4 or IFT74-mNeonGreen were seeded into glass-bottom 8-well chambers (CellVis, USA) and serum-starved for 24 h or 48 h, respectively. On the day of the FRAP experiments, cells were washed and incubated for at least 1 h at 37 °C with 5% CO_2_ in FluoroBrite medium supplemented with 1 μM SiR-Tubulin (Spirochrome, SC002) to visualize centrosomes or cilia. Prior to live-cell imaging, 20 mM HEPES was added to the medium. FRAP measurements were performed using a Leica TCS SP8 confocal microscope equipped with an oil immersion objective HC Plan-Apochromat 63 × NA 1.4 oil, CS2 at room temperature. Data acquisition was performed in 512 × 512-pixel format with pinhole 2.62 Airy, at a speed of 1000 Hz (BBS4) or 400 Hz (IFT74) in bidirectional mode and 8-bits resolution. Photobleaching of satellites (0.3 s) was performed with a circular spot (1 µm in diameter), and of ciliary tip (0.65 s) with a rectangle roi (~ 1 µm × 1 µm) at 100% intensity using a 20 mW 488 nm solid-state laser. Fluorescence recovery was monitored at low laser intensity (3–10%) at 0.26 s (satellites) or 0.648 s (ciliary tip) intervals until reaching the plateau of recovery, in total for 60 to 80 s after photobleaching. A total of 30–50 separate FRAP measurements were performed for each sample. All FRAP curves were normalized to fluorescence loss during acquisition following the subtraction of background fluorescence. Curve fitting was performed in GraphPad Prism software using the one-phase association fit. All individual curves were fitted at once to obtain the mean and 90% or 95% confidence intervals of the desired parameters, halftime T_1/2_ and the mobile fraction F_m_. Since the initial recovery after bleaching is contaminated by the diffusion, for simplicity and to estimate only the halftime of the protein fraction bound at the analyzed compartments, we restricted fitting of our data to times t > 2 s as we did in our previous studies [9, 41].

### Kymographs, data processing, and analysis

Cells expressing IFT74-mNeonGreen were seeded into glass-bottom 8-well chambers (CellVis, USA) and serum-starved for 48 h. On the day of the experiments, cells were washed and incubated for at least 1 h at 37 °C with 5% CO_2_ in FluoroBrite medium supplemented with 1 μM SiR-Tubulin (Spirochrome, SC002) to visualize centrosomes or cilia. Prior to live-cell imaging, 20 mM HEPES was added to the medium. Kymograph measurements were performed using a Zeiss Elyra 7 superresolution microscope equipped with an oil immersion objective α Plan-Apochromat 100x/1,46 Oil DIC M27 at room temperature. Data acquisition was performed in 1280 × 1280-pixel format and 16-bits resolution. Imaging was performed in frame interval of 250 ms for 300 frames in TIRF acquisition setting using angle of 66,93°. A total of 30 separate cilia measurements were performed for each sample. Individual IFT traces were analyzed manually using Fiji ImageJ according to Binó et al. [40] and the specific parameter values (event frequency, IFT velocity) were plotted in GraphPad Prism software.

#### Statistical analysis

Statistical analysis was performed using GraphPad Prism Version 5.04. The following statistical tests were used and are indicated in the respective Figure legends: Mann–Whitney test, Paired t-test and Spearman correlation.

## Results

### Pathogenic BBS1 patient mutations selectively disrupt the expression of BBSome subunits

**Fig. 1:**
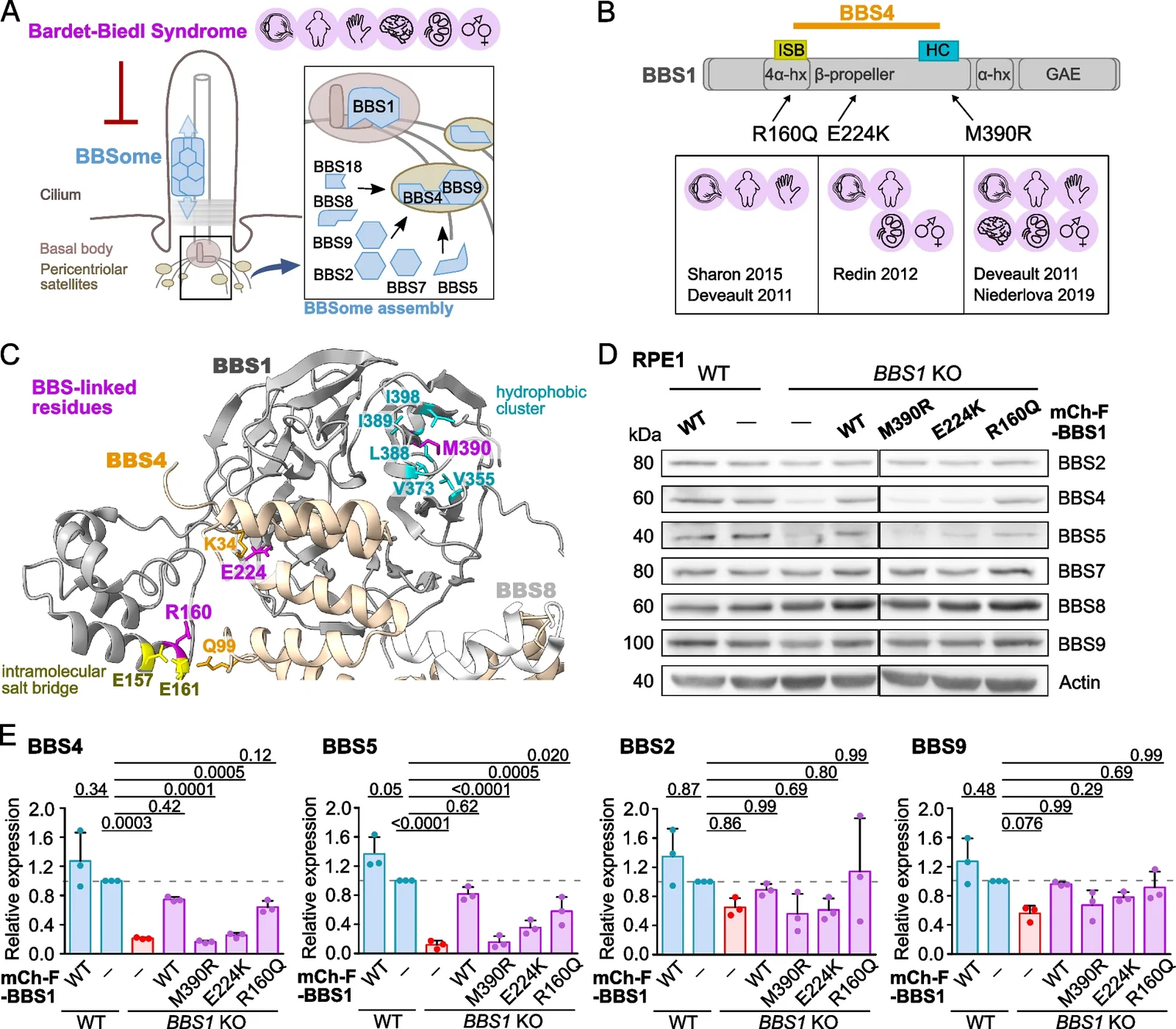
BBS linked BBS1 variants differentially alter expression of the BBSome subunits. **A** Schematic of the octameric BBSome complex, a ciliary cargo adaptor assembled at pericentriolar satellites and the basal body with the aid of BBS4 and BBS1, respectively. BBSome dysfunction leads to Bardet–Biedl syndrome, a multisystemic ciliopathy with variable tissue-specific symptom penetrance (affected organs indicated by icons). **B** Schematic of the BBS1 subunit of the BBSome showing its domain organization and the locations of BBS–associated mutations of varying clinical severity (indicated by organ-specific icons and respective studies). Based on structural analyses, the mutations map to the BBS1–BBS4 interaction interface (orange line). ISB, intramolecular salt bridge; HC, hydrophobic cluster; hx, α-helix. **C** Ribbon representation of the BBS1–BBS4 interaction interface from the cryo-EM structure of the human BBSome [12], shown together with a portion of BBS8 (white). The N-terminal β-propeller of BBS1 is shown in grey and the N-terminal TPR domain of BBS4 in orange; BBS-linked residues in BBS1 are highlighted in magenta. M390 of BBS1 is located in blade 7 of the β-propeller and is buried within a hydrophobic cluster formed by V355, V373, L388, I389, and I398 (cyan), distal from the BBS1–BBS4 interface. E224 of BBS1 lies at the BBS1–BBS4 interface in blade 4 of the β-propeller and is positioned near K34 of BBS4 (orange). R160 is located within the 4α-helical insert of the BBS1 β-propeller, where it forms an intramolecular salt bridge with E161 and E157 (yellow); this helical insert contacts the TPR domain of BBS4, with the R160 backbone positioned near Q99 of BBS4 (orange). Highlighted residues are shown as sticks. **D** Expression levels of endogenous BBSome subunits in parental and BBS1 variants expressing WT and *BBS1* KO RPE1 cell lines. Equal protein amounts were loaded into each lane. Actin is used as the loading control. Representative blots out of three independent experiments are shown. **E** Bar graphs depicting the relative expression levels of the endogenous BBSome subunits in parental and BBS1 variants expressing WT and *BBS1* KO RPE1 cell lines. Protein amounts were quantified using the Fiji ImageJ. Protein expression was first normalized to actin and then to levels in parental WT cell line. Average and SD of three independent experiments is shown. Statistical significance was calculated using one-way ANOVA followed by Tukey`s multiple comparisons test

Mutations in the BBS1 subunit of the BBSome are associated with comparatively milder Bardet–Biedl syndrome (BBS) phenotypes than mutations in core BBSome subunits BBS2, BBS7, and BBS9 [6]. Our work, along with that of others, has shown that loss of BBS1 results in accumulation of the pre-BBSome at pericentriolar satellites, whereas loss of other BBSome subunits disrupts pre-BBSome nucleation at these sites (Fig. 1A) [9, 29]. To investigate the pathogenic mechanisms by which BBS1 mutations contribute to BBS, we selected three variants with varying clinical penetrance: M390R, E224K, and R160Q (Fig. 1B). These mutations map to distinct structural regions of BBS1, enabling analysis of region-specific effects on BBSome assembly and function (Fig. 1B-C) [12, 30]. The M390R mutation lies in a buried hydrophobic cluster and introducing a charged arginine at this position destabilizes the local β-propeller packing, consistent with earlier reports showing loss of ARL6–GTP binding, and diminished BBS4 interaction [28, 36]. The E224K mutation lies at the BBS1–BBS4 interface and introduces a charge inversion that is likely to weaken the interaction by disrupting local electrostatic complementarity. The R160Q variant could disrupt an intramolecular salt bridge within the flexible 4α-helical region that contacts BBS4, increasing local flexibility and potentially loosening this interface.

To characterize the role of these mutations in the BBSome biology, we generated BBS1 variants tagged at the N-terminus with mCherry and FLAG (mCh-F-BBS1). These constructs were introduced into RPE1 *BBS1* knockout (KO) cells [9]. Since the M390R and R160Q mutations have been associated with protein degradation in BBS patients [10, 37], we first examined their protein expression in our model. We sorted cells double positive for mCherry and Thy1.1, a marker encoded in a bicistronic mCh-F-BBS1 ~ IRES-Thy1.1 transcript in the pMSCV vector (Fig. S1A). Correlation analysis of mCherry and Thy1.1 levels indicated that the mCh-F-BBS1 variants are stable and show no detectable mutation-induced degradation (Fig. S1A-B). Further analysis of BBS1 levels showed that both WT and variant proteins were expressed at levels approximately 40-fold higher than endogenous BBS1 (Fig. S1C-D). This allowed us to study their effects on BBSome assembly and function without interference from potential variant instability or degradation.

Our previous study demonstrated that deletion of BBS1 reduces the expression of several BBSome subunits—most prominently BBS4 and BBS5, and to a lesser extent BBS2, BBS7, BBS8, and BBS9—owing to impaired BBSome assembly [9]. We confirmed this observation and detected reduced levels of BBS4 and BBS5, whereas BBS8 and BBSome core subunits BBS2, BBS7 and BBS9 were largely not downregulated (Fig. 1D-E, S1E). This may indicate the formation of subcomplexes, such as the core BBSome and pre-BBSome, which could stabilize the interacting subunits [9, 10]. Expression of mCh-F-BBS1 WT restored BBSome subunit levels in the *BBS1* KO background (Fig. 1D-E, S1E). In contrast, the M390R and E224K variants failed to rescue the reduced subunit expression, whereas the R160Q variant unexpectedly restored expression nearly to WT levels (Fig. 1D-E, S1E). Together, these results indicate that BBS-linked BBS1 mutations differentially influence the expression of BBSome subunits.

### BBS1 variants impair GPR161 export from cilia during Sonic Hedgehog signaling

**Fig. 2:**
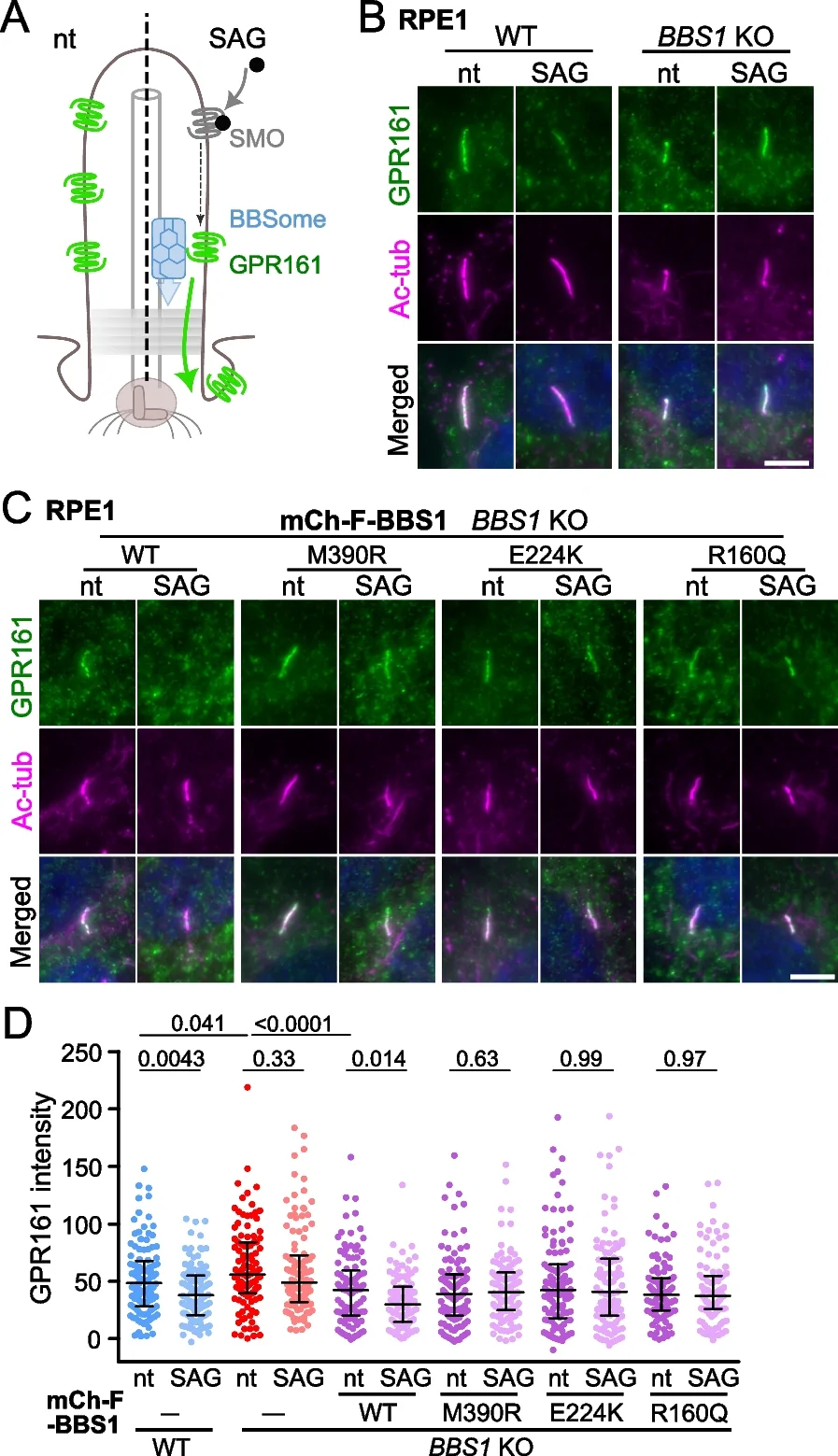
BBS1 variants impair GPR161 export from cilia during Sonic Hedgehog signaling. **A** Schematic of ciliary GPR161 localization in basal (non-treated) conditions and after Smoothened agonist (SAG) stimulation. In basal conditions, GPR161 is enriched in cilia, whereas SAG activation triggers BBSome-dependent export of GPR161 from the cilium. Representative micrographs of GPR161 in cilia in the WT and *BBS1* KO cell lines (**B**) and BBS1 variants expressing *BBS1* KO cell lines (**C**) starved for 24 h upon treatment with 200 nM SAG for 2 h. Antibody against acetylated tubulin (Ac-tub) was used to stain cilia. Nucleus was stained with 4′,6-diamidino-2-phenylindole (DAPI). Scale bar, 5 µm. **D** Intensity quantification of GPR161 in cilia in (**B**) and (**C**). Analysis was carried out using the Fiji ImageJ. Medians with interquartile range from three independent experiments of (*n* = 110–140 cilia). Statistical significance was calculated using two-tailed Mann–Whitney test

Next, we examined how these variants affect BBSome function in cilia. We assessed BBSome-mediated GPCR retrieval during signaling by focusing on the canonical BBSome cargo GPR161, an inhibitory GPCR in the Hedgehog (HH) pathway [42]. While GPR161 is present in cilia under steady-state conditions, ligand-induced triggering of the HH pathway leads to BBSome-dependent removal of GPR161 from cilia, thereby enabling pathway activation [15, 18, 29, 43] (Fig. 2A). Consistently, induction of the HH pathway using SAG (Smoothened agonist) reduced ciliary GPR161 signal under WT conditions, whereas GPR161 accumulated in cilia in *BBS1* KO background (Fig. 2B). Expression of mCh-F-BBS1 WT in *BBS1* KO cells restored GPR161 export from cilia upon SAG treatment (Fig. 2C-D). In contrast, all three BBS1 variants—M390R, E224K and R160Q—failed to restore GPR161 export in the *BBS1* KO background, demonstrating loss of function (Fig. 2C–D). Together, these data show that all BBS1 variants impair BBSome function during HH signaling.

### BBS1 variants differentially disrupt BBSome assembly and ciliary localization

**Fig. 3:**
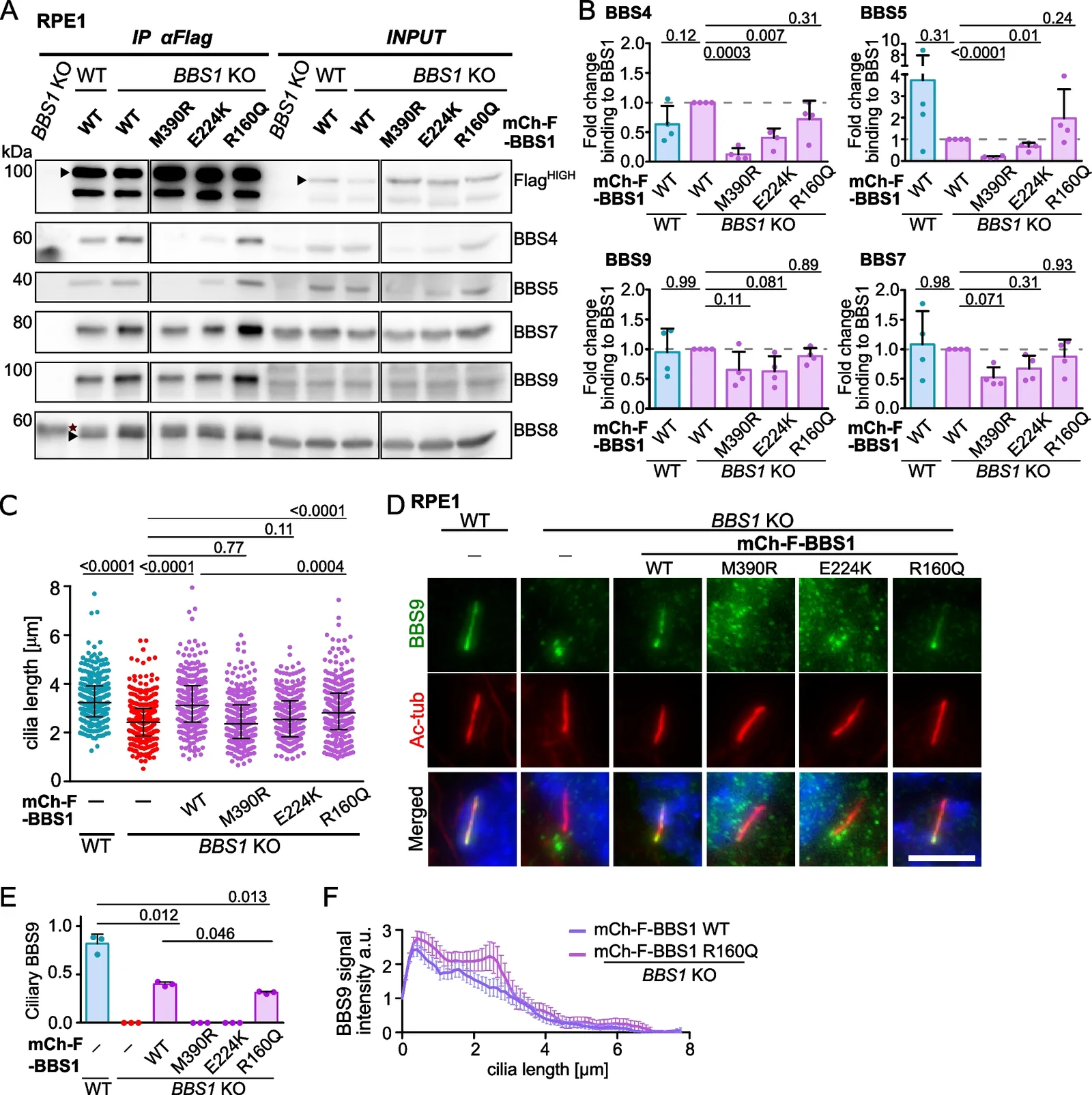
BBS1 variants differentially impact BBSome assembly and translocation to cilia. **A** Co-immunoprecipitation of the BBSome subunits from mCherry-FLAG tagged BBS1 variants expressing WT and *BBS1* KO RPE1 cell lines using anti-FLAG antibodies after 24 h serum starvation. Representative immunoblot out of four experiments is shown. **B** Quantification of the co-precipitated BBSome subunits in (**A**) was performed using the Fiji ImageJ software. Amount of the BBSome subunits was normalized to the levels of the mCherry-FLAG tagged BBS1 WT in *BBS1* KO RPE1 cell line detected on the respective membrane. Mean with SD of four experiments is shown. Statistical significance was calculated using one-way ANOVA followed by Tukey`s multiple comparisons test. **C** Quantification of cilia length in the parental and BBS1 variants expressing WT and *BBS1* KO RPE1 cell lines upon 24 h serum starvation. Antibody against acetylated tubulin (Ac-tub) was used to stain the primary cilia. Analysis was carried out using the Fiji ImageJ. Medians with interquartile range from three independent experiments of (*n* = 280–295 cilia). Statistical significance was calculated using two-tailed Mann–Whitney test. **D** Representative micrographs depicting BBS9 localization in the parental and BBS1 variants expressing WT and *BBS1* KO RPE1 cell lines upon 24 h serum starvation. Antibody against acetylated tubulin (Ac-tub) was used to stain the primary cilia. Nucleus was stained with 4′,6-diamidino-2-phenylindole (DAPI). Scale bar, 5 µm. **E** Quantification of ciliary BBS9 in (**D**). Analysis was carried out using the Fiji ImageJ. Mean and SD from three independent experiments (*n* = 160–230 cilia) is shown. Statistical significance was calculated using two-tailed paired t-test. **F** Distribution of BBS9 signal along the axoneme in *BBS1* KO RPE1 cells expressing BBS1 WT and R160Q variants in (**D**). Quantification was performed using the Fiji ImageJ. The plot shows mean and SD from n = 41 cilia for WT and n = 35 cilia for the R160Q variant

The loss-of function phenotype of mutations commonly associated with BBS suggests a defect in BBSome assembly [6, 9]. To assess how the BBS1 variants affect BBSome formation, we performed FLAG-based immunoprecipitation assays (Fig. 3A, S2A). The mCh-F-BBS1 R160Q variant co-precipitated BBSome subunits to comparable levels to the BBS1 WT. This suggests that the R160Q mutation does not impair BBS1 integration into the BBSome complex. In contrast, the mCh-F-BBS1 M390R and E224K variants showed markedly reduced association with the BBS4 and BBS5 subunits and retained substantial interactions with the BBSome core components (BBS7 and BBS9), as well as with the BBS8 subunit (Fig. 3A-B, S2A).

The fully assembled BBSome localizes to cilia, which is essential for its function and cilia length maintenance [9, 11, 18, 21]. First, we examined the effects of the BBS1 WT and variants on the cilia frequency and length using immunofluorescence (Fig. 3C-D, S2B-C). Loss of BBS1 lead to decreased cilia length in comparison to WT cells as observed before (Fig. 3C) [9, 18]. Expression of BBS1 WT in *BBS1* KO cells restored cilia length to that of WT cells. In contrast, expression of the BBS1 R160Q variant led to a partial rescue and expression of the BBS1 M390R or E224K variants failed to rescue the cilia length defect in *BBS1* KO cells (Fig. 3C). Next, we assessed the effects of mCh-F-BBS1 variants on BBSome ciliary targeting using BBS9 and mCherry signals as proxies (Fig. 3D, S2C). As a core BBSome subunit required for complex assembly, BBS9 serves as a reliable proxy of BBSome ciliary localization [9]. Consistent with previous observations, loss of BBS1 abolishes ciliary targeting of BBSome subunits, including BBS9, leading to their retention at pericentriolar satellites near the ciliary base (Fig. 3D-E) [9, 29]. Expression of either the mCh-F-BBS1 WT or R160Q variant restored BBS9 localization to cilia at comparable frequencies and with a comparable distribution along the cilia, (Fig. 3D-F). Likewise, mCh-F-BBS1 WT or R160Q localized to cilia at similar frequencies (Fig. S2C-D). In contrast, neither BBS9 nor mCh-F-BBS1 localized to cilia in *BBS1* KO cells expressing the mCh-F-BBS1 M390R or E224K variants (Fig. 2D-E, S2C-D).

Collectively, these findings suggest that BBS-linked mutations in BBS1 can disrupt specific steps of BBSome assembly and function without fully abolishing complex formation. Notably, the R160Q variant does not impair BBSome entry into cilia, indicating that BBS pathology may arise from defect in BBSome function within the cilium.

### E224K and M390R mutations in BBS1 disrupt pre-BBSome formation at pericentriolar satellites

At pericentriolar satellites, BBS4 together with BBS9 recruits BBSome subunits to initiate pre-BBSome assembly. In the presence of BBS1, transition of the pre-BBSome into the mature BBSome and its subsequent translocation to the cilium reduces the pool of BBSome subunits at satellites. In contrast, in the absence of BBS1, the pre-BBSome forms but remains retained at pericentriolar satellites [9].

**Fig. 4:**
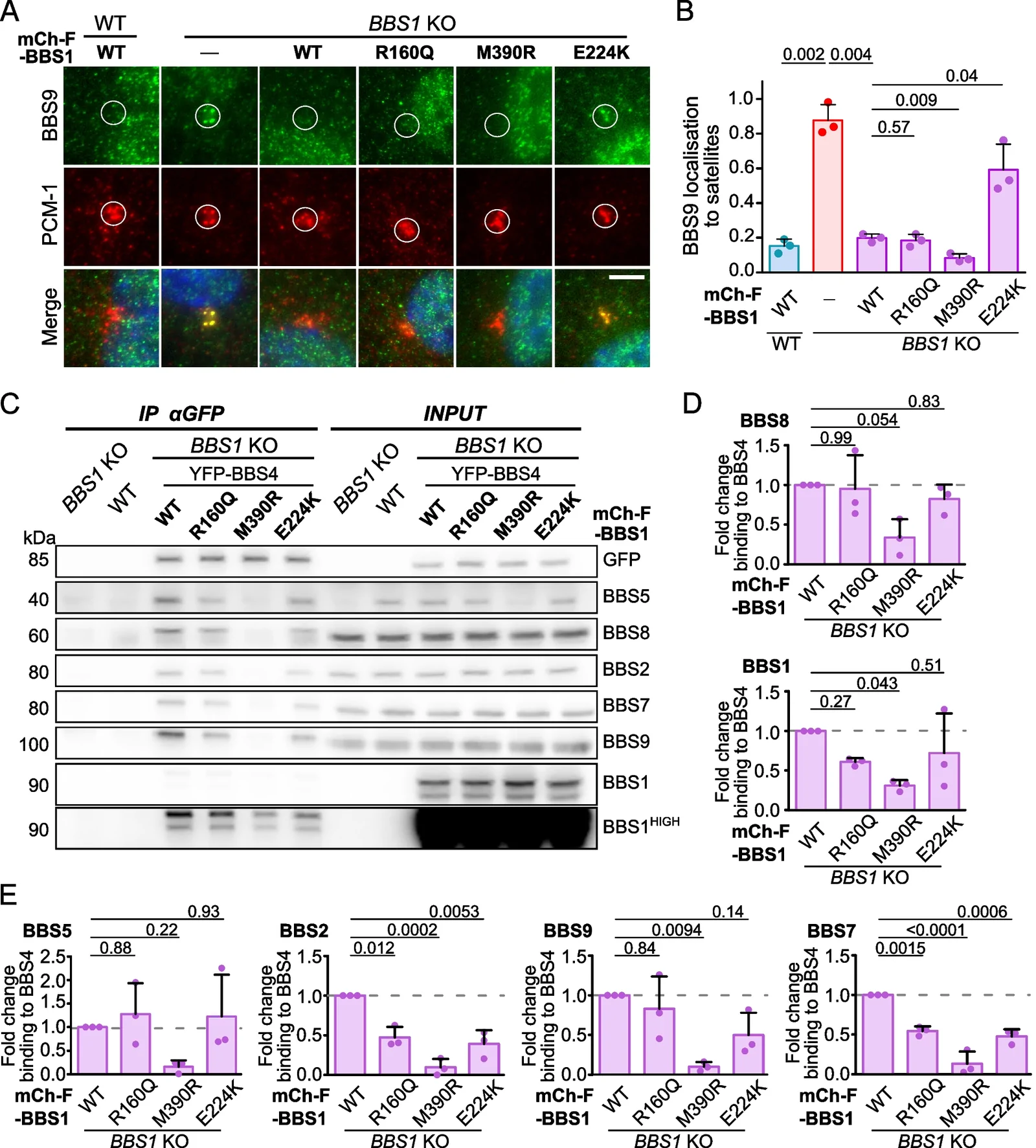
BBS1 E224K and M390R alter the pre-BBSome assembly at pericentriolar satellites. Representative micrographs (**A**) and quantification (**B**) showing localization of BBS9 in pericentriolar satellites in the parental and BBS1 variants expressing WT and *BBS1* KO RPE1 cell lines. Antibody against PCM-1 was used to stain the satellites. Nucleus was stained with 4′,6-diamidino-2-phenylindole (DAPI). Scale bar, 5 µm. Analysis was carried out using the Fiji ImageJ software. Mean and SD from three independent experiments (*n* = 220–340 satellites). Statistical significance was calculated using two-tailed paired t-test. **C** Co-immunoprecipitation of the BBSome subunits from YFP-BBS4 and BBS1 variants expressing *BBS1* KO RPE1 cell lines using anti-GFP antibodies after 24 h serum starvation. Representative immunoblot out of three experiments is shown. Quantification of the co-precipitated BBS8 and BBS1 (**D**) and BBS5 and BBSome core subunits (**E**) in (**C**) was performed using the Fiji ImageJ. Amount of the BBSome subunits was normalized to the YFP-BBS4 levels in the mCherry-FLAG tagged BBS1 WT expressing *BBS1* KO RPE1 cell line, as detected on the respective membrane. Mean with SD of three experiments is shown. Statistical significance was calculated using one-way ANOVA followed by Tukey`s multiple comparisons test

Our data show that the M390R and E224K mutations in BBS1 alter its interaction with BBS4 to different extent (Fig. 3A-B). To assess the impact of BBS1 variants on BBS4-driven pre-BBSome assembly at satellites, we analyzed BBS9 co-localization with PCM-1, a satellite marker (Fig. 4A). In the presence of R160Q variant, BBS9 was devoid from satellites to a similar extent as with the WT variant, in both *BBS1* KO and WT backgrounds (Fig. 4B), consistent with full BBSome assembly and translocation to cilia under these conditions (Fig. 3). Interestingly, the E224K variant promoted BBS9 accumulation at satellites to levels comparable to those in the *BBS1* KO condition, indicating formation of the pre-BBSome (Fig. 4B). By contrast, in the presence of M390R variant, BBS9 localized to satellites to an even lesser extent compared to WT and R160Q conditions (Fig. 4A-B). Since the M390R variant associates with BBS9, BBS7 and BBS8, but not with BBS4 and BBS5 subunits (Fig. 3A-B, S2A), these data suggest that this subcomplex may be depleted from satellites into the cytoplasm.

To explore this further, we expressed YFP-tagged BBS4 in the cells and used YFP-based immunoprecipitation to pull out the complexes (Fig. 4C-E, S3A). Because YFP-BBS4 mainly localizes to the centrosome-associated satellites in all BBS1-variant cell lines (Fig. S3A), the isolated complexes reflected mainly interactions happening at those sites. The WT, R160Q and E224K variants supported pre-BBSome/BBSome complex formation, with BBS4 efficiently binding BBS8, BBS1, BBS5 and substantial amounts of the core BBSome components (Fig. 4D-E). The E224K mutation enabled incorporation of BBS1 into the pre-BBSome, and this E224K-BBSome complex was retained at satellites (Fig. 4A-D). We previously showed that, BBS4 dissociates faster from satellites in the absence of BBS1, reflecting pre-BBSome instability [9]. Using FRAP, we found that BBS4 dissociation in the presence of BBS1 E224K is comparable to WT, indicating that the E224K-BBSome is neither destabilized nor sequestered at satellites (Supp. Figure 3B–D). These results show that the BBS1 E224K mutation allows BBSome assembly but specifically impairs its translocation to cilia (Fig. 3D-E), likely due to compromised complex integrity (Fig. 1C).

In the presence of the BBS1 M390R variant, BBS4 bound only minimal amounts of BBS1 and BBS8, and virtually no BBS5 or core BBSome components BBS2, BBS7, and BBS9 (Fig. 4D–E). Despite this, the BBSome core still associated directly with BBS1 M390R (Fig. 3A–B), while BBS9 levels at satellites remained low (Fig. 4B), suggesting that BBS1 M390R sequesters the core away from BBS4 and satellites, preventing pre-BBSome nucleation. In contrast, BBS1 knockout cells showed clear enrichment of BBS9 and other subunits at satellites (Fig. 4A-B) [9], and modestly increased BBS4 association with BBS9 and BBS7 (Supp. Figure 3E–F).

Altogether, these data suggest that E224K behaves as a hypomorphic allele, with a milder phenotype than *BBS1* KO, likely due to a fraction of partially functional BBSome at the satellites. In contrast, M390R more closely resembles *BBS1* KO, as it interferes with pre-BBSome formation, albeit via a distinct mechanism.

### BBS1 R160Q variant impairs retrieval of TOM1L2-bound ubiquitinated cargoes from cilia

**Fig. 5:**
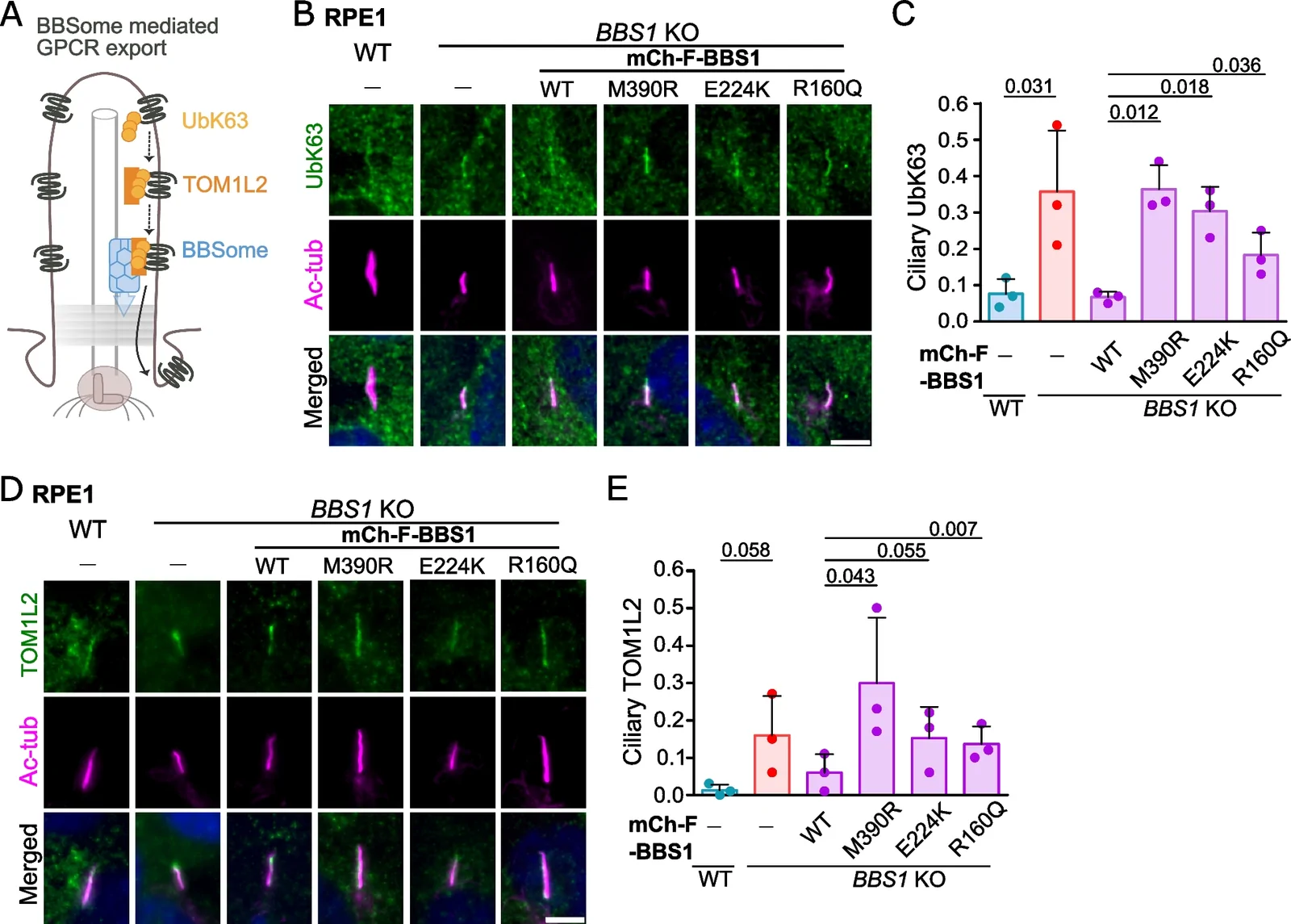
BBS1 variants impair retrieval of ubiquitinated cargo-TOM1L2 complexes from cilia. **A** Schematic of ubiquitinated ciliary cargo recognition by the adaptor TOM1L2, which mediates BBSome-dependent export of these cargoes from cilia. Representative micrographs (**B**) and frequency quantification (**C**) of UbK63 in cilia in the parental and BBS1 variants expressing WT and *BBS1* KO RPE1 cell lines starved for 48 h. Antibody against acetylated tubulin (Ac-tub) was used to stain cilia. Nucleus was stained with 4′,6-diamidino-2-phenylindole (DAPI). Scale bar, 5 µm. Analysis was carried out using the Fiji ImageJ. Means with SD from three independent experiments of (*n* = 150–210 cilia) are shown. Statistical significance was calculated using one-tailed paired t-test. Representative micrographs (**D**) and frequency quantification (**E**) of TOM1/2L in cilia in the parental and BBS1 variants expressing WT and *BBS1* KO RPE1 cell lines starved for 48 h. Antibody against acetylated tubulin (Ac-tub) was used to stain cilia. Nucleus was stained with 4′,6-diamidino-2-phenylindole (DAPI). Scale bar, 5 µm. Analysis was carried out using the Fiji ImageJ. Means with SD from three independent experiments of (*n* = 290–350 cilia) are shown. Statistical significance was calculated using one-tailed paired t-test

Our data show that BBS-linked BBS1 mutations impair BBSome function through distinct mechanisms: M390R disrupts BBSome assembly, E224K impairs BBSome transport to cilia, whereas R160Q does not perturb BBSome assembly or ciliary entry, suggesting a defect in BBSome function within cilia. A key role of the ciliary BBSome is the removal of cargoes marked by K63-linked ubiquitin through the ubiquitin adaptor TOM1L2 (Fig. 5A) [15, 16]. We therefore analyzed ciliary UbK63 and TOM1L2 levels by immunofluorescence (Fig. 5B, D) to test whether the R160Q variant impairs BBSome association with TOM1L2-bound cargoes, thereby disrupting their export from cilia.

As reported previously, UbK63 and TOM1L2 accumulate in cilia of cells lacking the BBSome [16]. Consistent with this, we observed increased abundance of ciliary UbK63 and TOM1L2 in *BBS1* KO cells compared to WT cells and *BBS1* KO cells expressing the mCh-F-BBS1 WT protein (Fig. 5C, E). Similar increase of ciliary UbK63 and TOM1L2 were observed in *BBS1* KO cells expressing the BBS1 M390R or E224K variants, indicating that these variants phenocopy the absence of functional ciliary BBSome in *BBS1* KO cells (Fig. 5C, E). Notably, ciliary UbK63 was also elevated in *BBS1* KO cells expressing the mCh-F-BBS1 R160Q variant, although to a lesser extent than in cells expressing the M390R and E224K variants (Fig. 5B-C). In addition, ciliary TOM1L2 abundance also increased in these cells, indicating that BBSome–BBS1 R160Q complex fails to export the ubiquitinated TOM1L2-bound cargoes (Fig. 5D-E).

Collectively, these data show that the R160Q variant phenocopies the ciliary export defect observed in *BBS1* KO cells.

### BBS1 R160Q disrupts TOM1L2-GPCR coupling to IFT

**Fig. 6:**
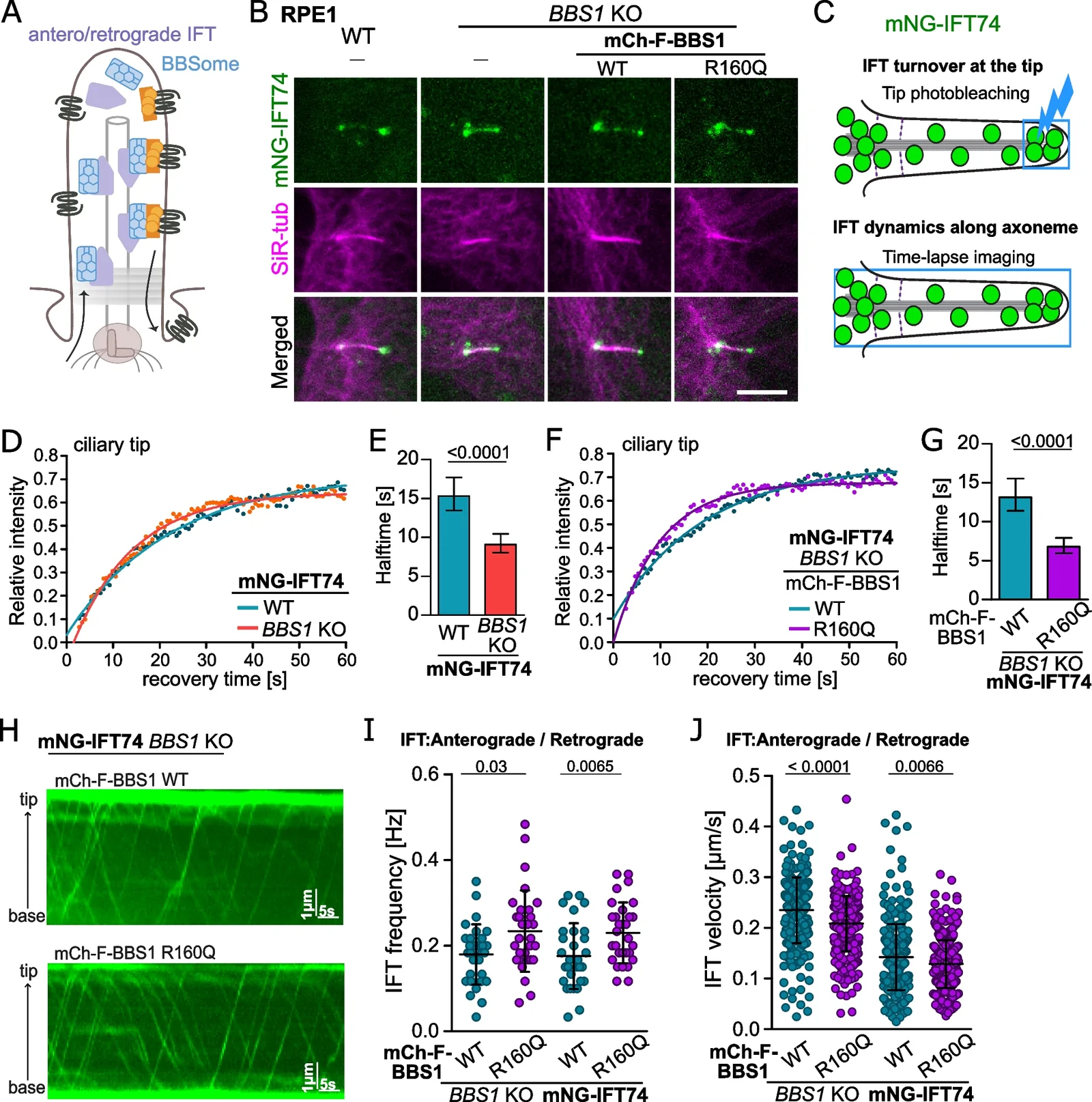
BBS1 R160Q disrupts TOM1L2-GPCR coupling to IFT. **A** Schematic of anterograde and retrograde IFT in cilia. At the ciliary tip, the BBSome and IFT complexes undergo remodeling to assemble retrograde IFT trains, which mediate the export of TOM1L2-associated cargoes. **B** Representative micrographs from the FRAP experiments depicting localization of mNeonGreen (mNG)-IFT74 in the parental and BBS1 WT and R160Q variants expressing WT and *BBS1* KO RPE1 cell lines. Ciliary axonemes and microtubules were stained with 1 μM SiR-tubulin. Scale bar, 5 µm. **C** Schematic representation of the FRAP (top) and kymograph (bottom) analyses of cilia-localized mNG-IFT74 (green circles). Ciliary tip (blue box) was photobleached for 0.65 s using a 20 mW 488 nm solid-state laser at 100% intensity (blue lightning bolt). Kymographs were generated from time-lapse images acquired across the entire cilium (blue rectangle). **D**, **F** FRAP analysis of the mNG-IFT74 in the parental and BBS1 WT and R160Q variants expressing WT and *BBS1* KO RPE1 cell lines upon 48 h serum starvation. Recovery curves were fitted at once using one phase association fit. Analysis was carried out using the GraphPad Prism. Means of (*n* = 45–50) measurements from four independent experiments are shown. **E**, **G** Bar graphs depicting recovery halftimes (s) derived from curve fits in (**D**, **F**). Error bars represent the 95% confidence interval and p-value is shown. **H** Representative kymograph images of mNG-IFT74 traces in BBS1 WT and R160Q expressing variants in *BBS1* KO RPE1 cell lines. Anterograde IFT trajectories move from the ciliary base to tip (bottom-to-top direction); retrograde trajectories move from tip to base (top-to-bottom direction). **I** Quantification of mNG-IFT74 anterograde and retrograde transport frequencies based on kymograph analyses as shown in (H). Analysis was carried out using the Fiji ImageJ. Means with SD from three independent experiments (*n* = 30 cilia) are shown. Statistical significance was calculated using two-tailed Mann–Whitney test. **J** Quantification of mNG-IFT74 anterograde and retrograde transport velocities based on (H). Means with SD from three independent experiments as in (**I**) are shown (*n* = 30 cilia: anterograde IFT traces n = 323–421, retrograde IFT traces n = 316–415). Analysis was carried out using the Fiji ImageJ. Statistical significance was calculated using two-tailed Mann–Whitney test

Since the BBS1 R160Q variant allows BBSome assembly but not cargo export, we further examined whether it disturbs TOM1L2-cargo loading onto IFT. Studies in olfactory neurons, *C. elegans*, and *Chlamydomonas* showed that the BBSome associates with the IFT machinery and regulates IFT dynamics and turnaround at the ciliary tip [14, 44–46], where anterograde trains are remodeled to initiate retrograde transport (Fig. 6A) [47]. First, we investigated how the BBSome regulates mammalian IFT dynamics by measuring the ciliary tip dwell time of mNeonGreen-IFT74 (mNG-IFT74) in WT and *BBS1* KO cells using fluorescence recovery after photobleaching (FRAP) approach (Fig. 6B-C). Since IFT trains pause and remodel at the ciliary tip before engaging retrograde motors and cargoes, measuring their dwell time (halftime of recovery) serves as an indirect readout of retrograde transport initiation. In *BBS1* KO cells, mNG-IFT74 fluorescence at the ciliary tip recovered significantly faster (half-time, T₁/₂ ≈ 9.1 s) compared with WT cells (T₁/₂ ≈ 15.3 s) (Fig. 6D-E). The shorter dwell time of IFT in the absence of the BBSome likely reflects a failure to engage BBSome-dependent cargoes, resulting in earlier initiation of retrograde transport. Introduction of the BBS1 WT variant into mNG-IFT74 *BBS1* KO cells (Fig. 6B) restored IFT dwell time at the ciliary tip to comparable value (T₁/₂ ≈ 13.2 s) observed in WT cells (T₁/₂ ≈ 15.3 s) (Fig. 6F-G). In contrast, IFT dwell time at the ciliary tip decreased in cells expressing the BBS1 R160Q–containing BBSome (T₁/₂ ≈ 6.8 s), phenocopying the short dwell time observed in BBSome-deficient cells (T₁/₂ ≈ 9.1 s) (Fig. 6F-G). These data indicate that the BBS1 R160Q variant prevents IFT to load the TOM1L2-dependent cargoes, resulting in earlier departure of retrograde IFT trains.

Next, we employed kymograph-based analysis of IFT trajectories in cilia with comparable length to investigate the effects of the BBSome and BBSome-BBS1 R160Q on anterograde and retrograde IFT (Fig. 6C, H, S4A-B). Loss of the BBSome in *BBS1* KO cells did not alter anterograde or retrograde IFT frequencies compared to WT cells (Fig. S4C). However, both anterograde and retrograde transport velocities were increased, consistent with less constrained IFT movement in the absence of BBSome and TOM1L2-cargo loaded trains (Fig. S4D). In contrast, both anterograde and retrograde IFT frequencies were increased, while transport velocities were reduced, in cells expressing the BBSome containing BBS1 R160Q variant compared to BBS1 WT (Fig. 6I-J). These data suggest that IFT trains containing the BBSome-BBS1 R160Q undergo more frequent entry and recycling but are less efficiently engaged in productive long-range transport. As a result, IFT particles can cycle locally, leading to repeated engagement attempts at the ciliary tip, consistent with increased IFT turnover (Fig. 6F-G). Together, both conditions converge on impaired BBSome-dependent cargo handling at the ciliary tip, consistent with increased local IFT turnover associated with defective export function.

**Fig. 7:**
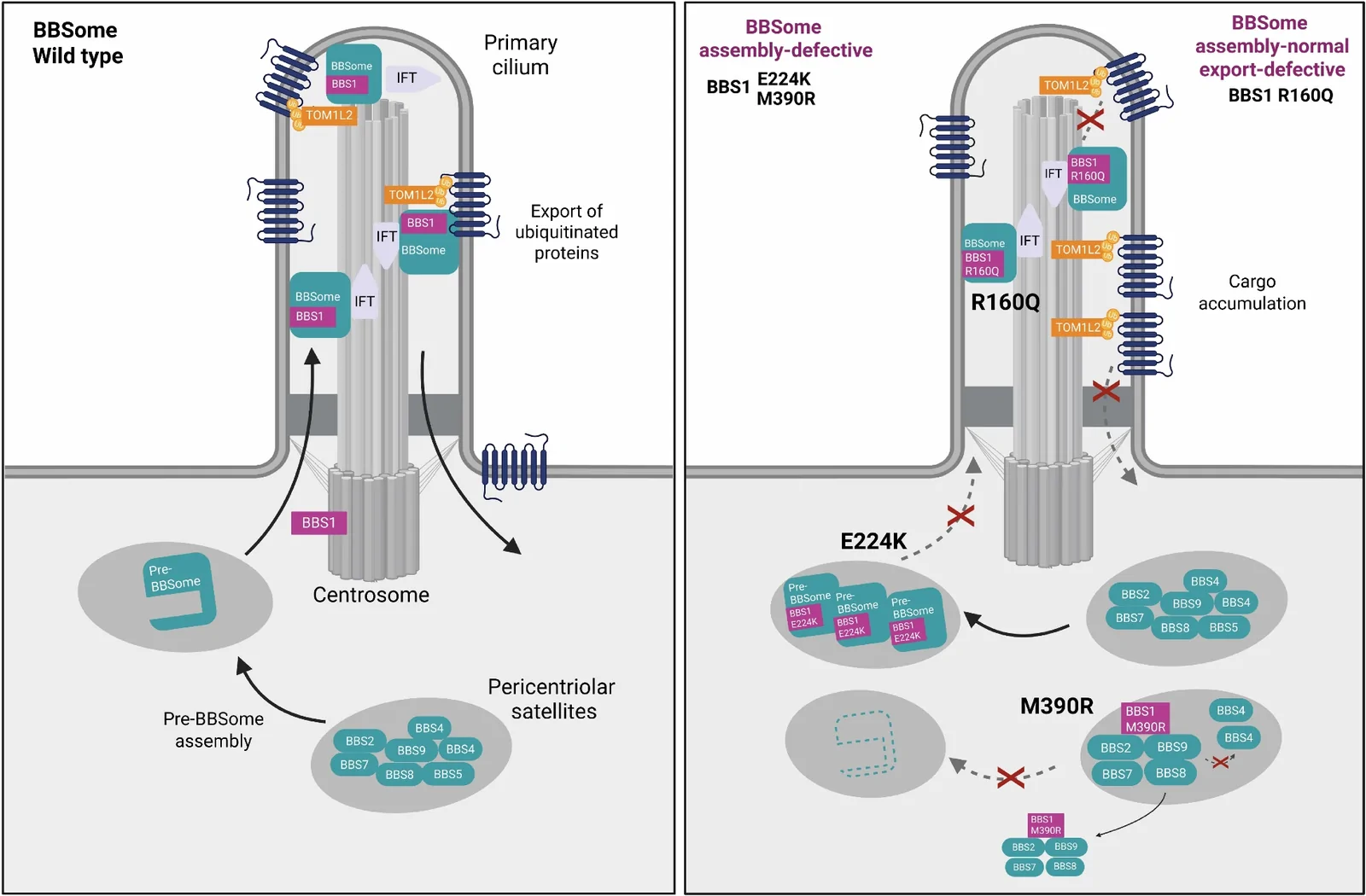
BBS1 mutation–specific defects in BBSome assembly and function define distinct pathogenic classes. In WT condition, BBS4 at pericentriolar satellites nucleates pre-BBSome assembly, which is completed by BBS1 to form a stable, mature BBSome that enters the cilium. Within cilia, the BBSome cooperates with IFT to mediate export of TOM1L2-marked, ubiquitinated cargoes. BBS1 mutations linked to Bardet–Biedl syndrome can be divided into two functional classes based on how they impair BBSome assembly and function in cilia. M390R and E224K belong to a class of mutations that disrupt distinct stages of BBSome assembly and maturation: M390R impairs initial pre-BBSome assembly at satellites, whereas E224K disrupts BBSome maturation and prevents its translocation from satellites to the cilium. In contrast, R160Q represents a second class of mutations that supports BBSome assembly and ciliary localization but impairs BBSome function within cilia. Specifically, R160Q prevents TOM1L2-marked cargo loading onto IFT, resulting in defective GPCR export and increased IFT turnover

Collectively, our results demonstrate that BBS1 mutations linked to BBS fall into distinct functional classes defined by their impact on BBSome assembly and ciliary function (Fig. 7). M390R and E224K disrupt BBSome assembly at different stages, impairing pre-BBSome formation and BBSome maturation/ciliary translocation, respectively. In contrast, R160Q permits BBSome assembly and ciliary localization but selectively disrupts BBSome-dependent TOM1L2-mediated cargo export via IFT.

## Discussion

Bardet–Biedl syndrome (BBS) is a ciliopathy characterized by striking clinical heterogeneity, even among individuals carrying mutations in the same gene [6, 27, 33, 34, 48]. Mutations in BBS1 are distributed throughout the gene without clear mutational hotspots, and missense variants generally cause milder disease than loss-of-function mutations (Beales et al., 2003; Chou et al., 2019; Niederlova et al., 2019). In this study, we investigated the molecular mechanisms underlying the pathogenicity of three BBS1 missense variants—M390R, E224K, and R160Q – displaying distinct levels of clinical penetrance [6, 32–35, 37].

The M390R mutation is the most prevalent variant among BBS patients (~ 80% of BBS cases) [6, 27]. Our data, together with previous studies, provide a mechanistic explanation for the pathogenic effects of the M390R mutation, demonstrating that it destabilizes the BBS1 β-propeller structure [28, 31], and weakens interactions with the BBS1 partners BBS4 and BBS5. These combined defects impair productive pre-BBSome assembly at pericentriolar satellites [9, 36]. Notably, the association of the BBS1 M390R variant with the BBSome core (BBS2/7/9) indicates that this mutation does not disrupt BBS chaperonin-mediated stabilisation of BBS2 and BBS7 [10, 49]. Instead, the BBS1 M30R variant prevents recruitment of the BBSome core to BBS4 and sequesters the core complex from satellites into the cytoplasm. At the cellular level, the M390R mutation approximates a functional BBSome-null state, explaining its broad clinical spectrum despite BBS1 being considered a “milder” BBS gene [6]. In addition, the M390R variant may exert further pathogenic effects through altered interactions with BBS4, a core satellite-associated protein [9, 50]. Dysregulation of BBS4 dynamics could disrupt satellite composition and homeostasis, impairing centrosomal protein trafficking, ciliogenesis, and the cell-cycle-related functions [5, 50–53]. Such defects likely exacerbate BBSome deficiency and underlie the severe BBS1 M390R phenotype.

In contrast, the E224K mutation represents an intermediate defect, producing sub-threshold BBS features that can lead to misdiagnosis as Alström syndrome or to an unconfirmed diagnosis due to limited evidence supporting its pathogenicity [34, 38]. The BBS1 E224K variant permits pre-BBSome assembly at satellites and associates with this complex; however, the resulting BBSome–BBS1 E224K fails to translocate to the cilium, likely via a BBS3-dependent mechanism [11, 30, 54]. Our data and structural analyses point to incomplete complex maturation, reflecting weakened integrity that may be critical for complex stability and activation [12, 30, 31, 54].

The R160Q substitution arises from a mutation affecting the 5′ splice site, which compromises splicing efficiency and leads to aberrant BBS1 mRNA processing [37, 55]. Although misspliced transcripts are expected to undergo nonsense-mediated decay, reduced levels of correctly spliced transcripts may lead to decreased BBS1 protein levels, potentially in tissue-specific manner. The R160Q variant has been linked to milder phenotypes or, in some cases, isolated non-syndromic retinitis pigmentosa [33–35, 37]. The R160Q elevates ciliary K63-linked ubiquitin and TOM1L2, indicating impaired BBSome–TOM1L2 recognition, a key step in BBSome-dependent retrieval of ubiquitinated cargoes from cilia [15, 16, 24]. The milder penetrance of R160Q mutation can be explained by the lower abundance of ciliary K63-linked ubiquitin and TOM1L2 in comparison to E224K, M390R and *BBS1* KO conditions. Comparison of the ciliary ubiquitinomes across these conditions may reveal altered protein turnover within specific ciliary pathways [56, 57], providing additional insight into the molecular basis of BBS severity. Although R160Q selectively impairs BBSome-dependent cargo export, it retains BBSome ciliary localization and other functions in IFT, as demonstrated by the partial restoration of cilia length. Moreover, TOM1L2-dependent retrieval may be particularly critical in photoreceptors, which rely on continuous protein and lipid turnover to maintain membrane homeostasis [23, 24, 58, 59], providing a rationale for the predominantly retinal phenotype associated with R160Q mutation.

To enter the cilium, the BBSome is loaded onto anterograde IFT trains at the ciliary base, functioning both as a cargo for IFT and as an adaptor for BBSome-dependent transmembrane ciliary proteins [11, 14, 44, 60, 61]. There is limited knowledge about how the BBSome regulates IFT dynamics in mammalian cilia, despite clear evidence supporting their genetic interaction in BBS patients and mouse models [17, 62]. In our study, we leveraged the BBS1 R160Q variant to provide the first insights into IFT behavior directly within cilia in the context of a BBS-linked mutated BBSome. Because BBSome-BBS1 R160Q still reaches cilia, its association with anterograde IFT trains is likely maintained [14, 60]. However, these trains cycle less efficiently, exhibiting increased IFT frequencies but reduced transport velocities. We propose that after remodeling at the ciliary tip, retrograde BBSome-BBS1 R160Q trains fail to efficiently load TOM1L2-marked cargo and return prematurely. This kinetic defect is compatible with cilia assembly and architecture but may impair dynamic ciliary trafficking and signaling, providing a potential explanation for the relatively mild R160Q phenotype.

Finally, it remains to be determined whether cilia-localized BBSome-BBS1 R160Q is sufficient to prevent other extraciliary defects associated with BBSome deficiency, including disrupted planar cell polarity and WNT signalling, increased extracellular vesicle release, mitochondrial dysfunction, and altered cell cycle progression [20, 50, 63, 64], which will define how BBSome dysfunction drives BBS pathology of varying severity.

## Conclusions

Our study shows that BBS1 mutations cause Bardet–Biedl syndrome through distinct molecular mechanisms that differentially disrupt BBSome function. We identify two functional classes of disease-associated variants. Mutations such as M390R and E224K impair BBSome assembly and maturation, preventing efficient ciliary entry and leading to severe trafficking defects. In contrast, the R160Q mutation preserves BBSome assembly and localization but selectively disrupts BBSome function within cilia by uncoupling TOM1L2-marked cargoes from IFT, resulting in defective GPCR export and increased IFT turnover.

These mutation-specific defects correlate with clinical severity, linking impaired BBSome assembly and trafficking to more penetrant disease and selective export failure to milder phenotypes. Together, our findings provide a mechanistic framework connecting BBS1 genotype to ciliary dysfunction and highlight the importance of functional stratification of BBS1 variants for diagnosis, prognosis, and therapeutic development in Bardet–Biedl syndrome.

## Supplementary Information

Supplementary Material 1.

Supplementary Material 2.

## Data Availability

This study includes no data deposited in external repositories.
