# Supplementary material for: Bardet-Biedl syndrome 1 mutations differentially impact BBSome integrity and ciliary trafficking
Source: Cell Commun Signal. 2026 Jun 19;24:462. doi: 10.1186/s12964-026-03006-8 (PMC13520375; doi:10.1186/s12964-026-03006-8)

Figure 1D

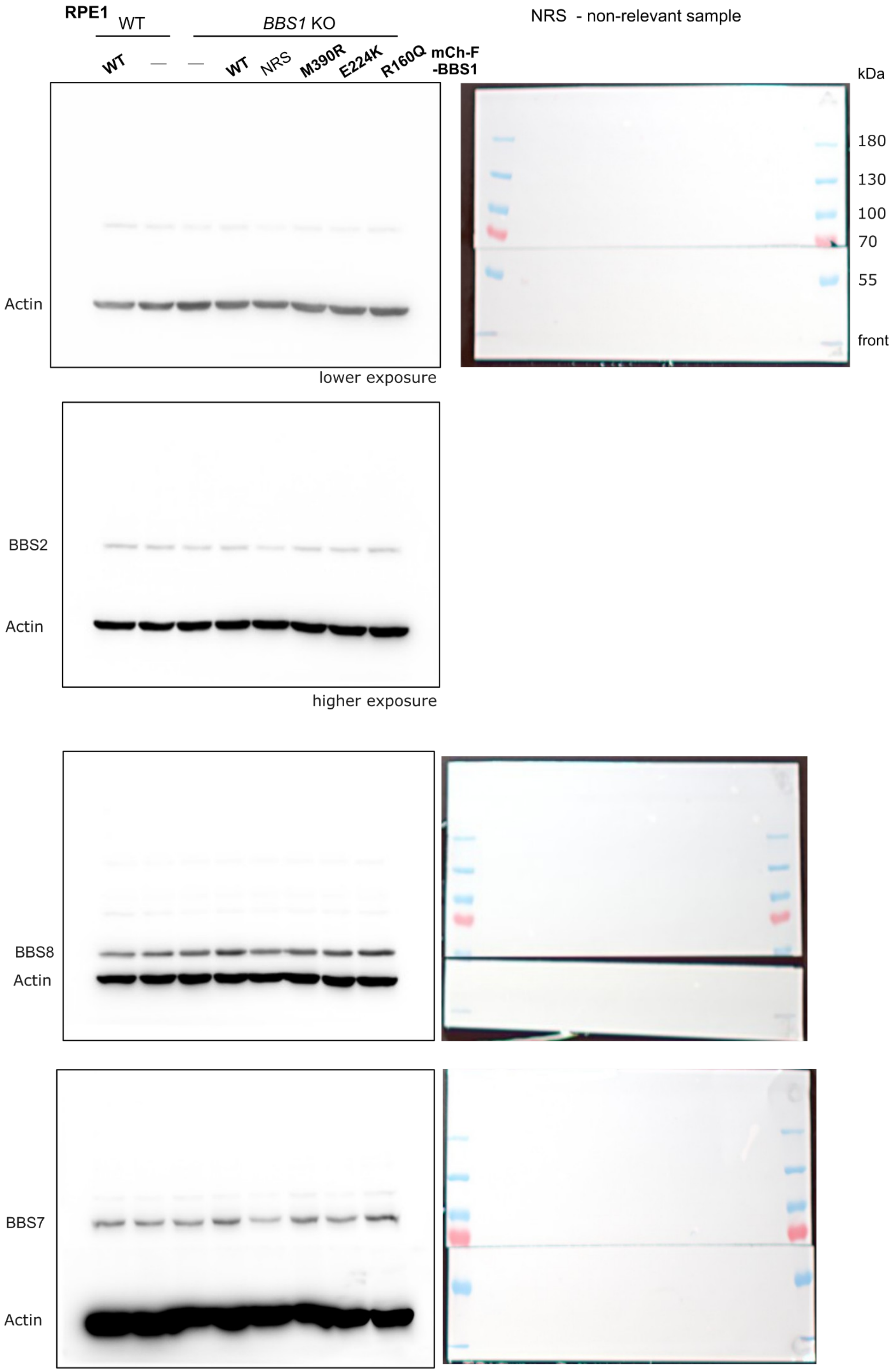
RPE1
WT
BBS1 KO
WT
—
—
WT
NRS
M390R
E224K
R160Q
mCh-F -BBS1
NRS - non-relevant sample
kDa
180
130
100
70
55
front
Actin
lower exposure
BBS2
Actin
higher exposure
BBS8
Actin
BBS7
Actin

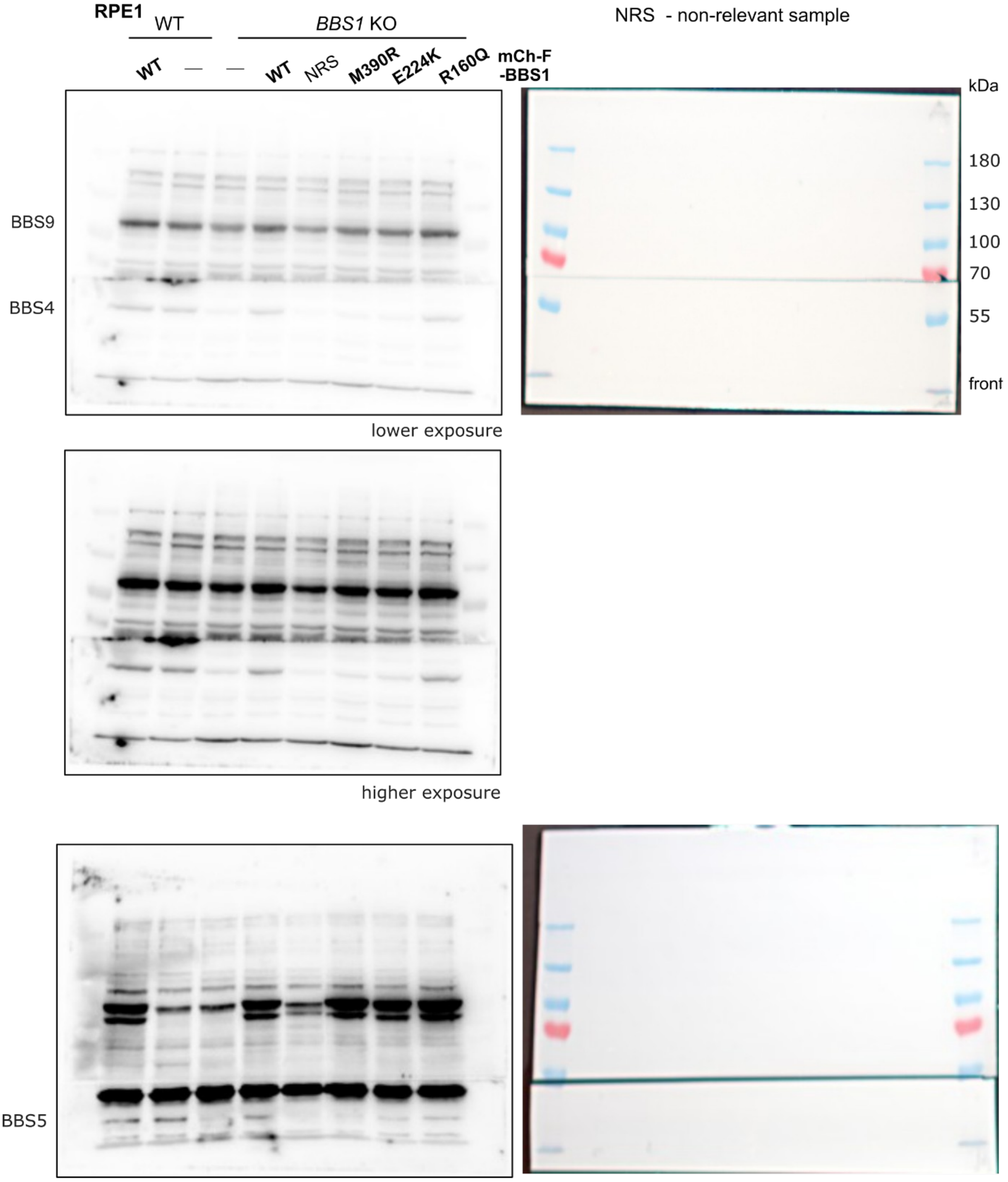
RPE1
WT
BBS1 KO
WT
—
—
WT
NRS
M390R
E224K
R160Q
mCh-F -BBS1
NRS - non-relevant sample
kDa
180
130
100
70
55
front
BBS9
BBS4
lower exposure
higher exposure
BBS5

Figure 3A

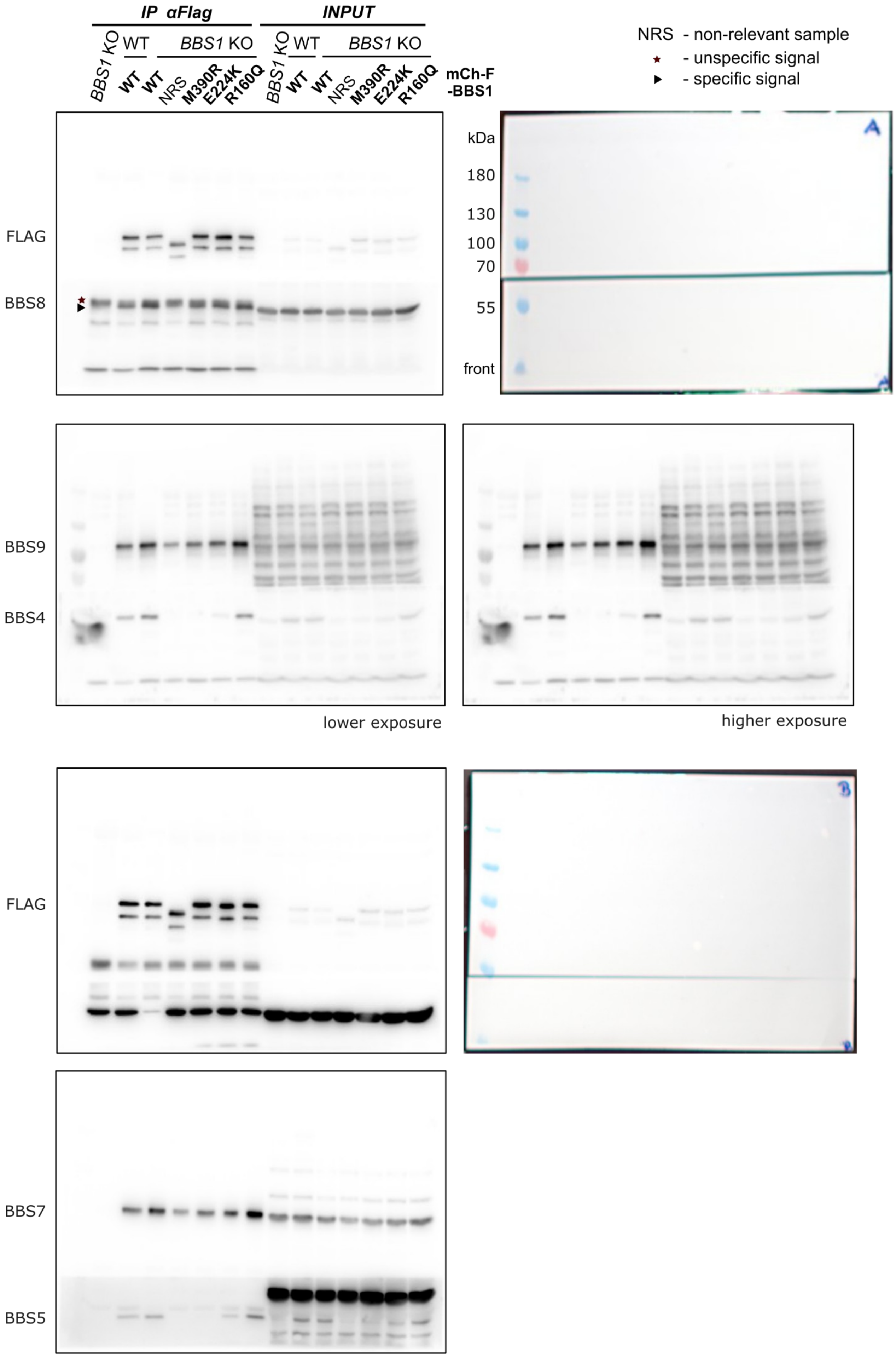
IP αFlag
INPUT
BBS1 KO
WT
BBS1 KO
WT
WT
NRS
M390R
E224K
R160Q
mCh-F
-BBS1
NRS - non-relevant sample
★ - unspecific signal
▶ - specific signal
FLAG
BBS8
kDa
180
130
100
70
55
front
A
BBS9
BBS4
lower exposure
higher exposure
FLAG
B
BBS7
BBS5

Figure 4C

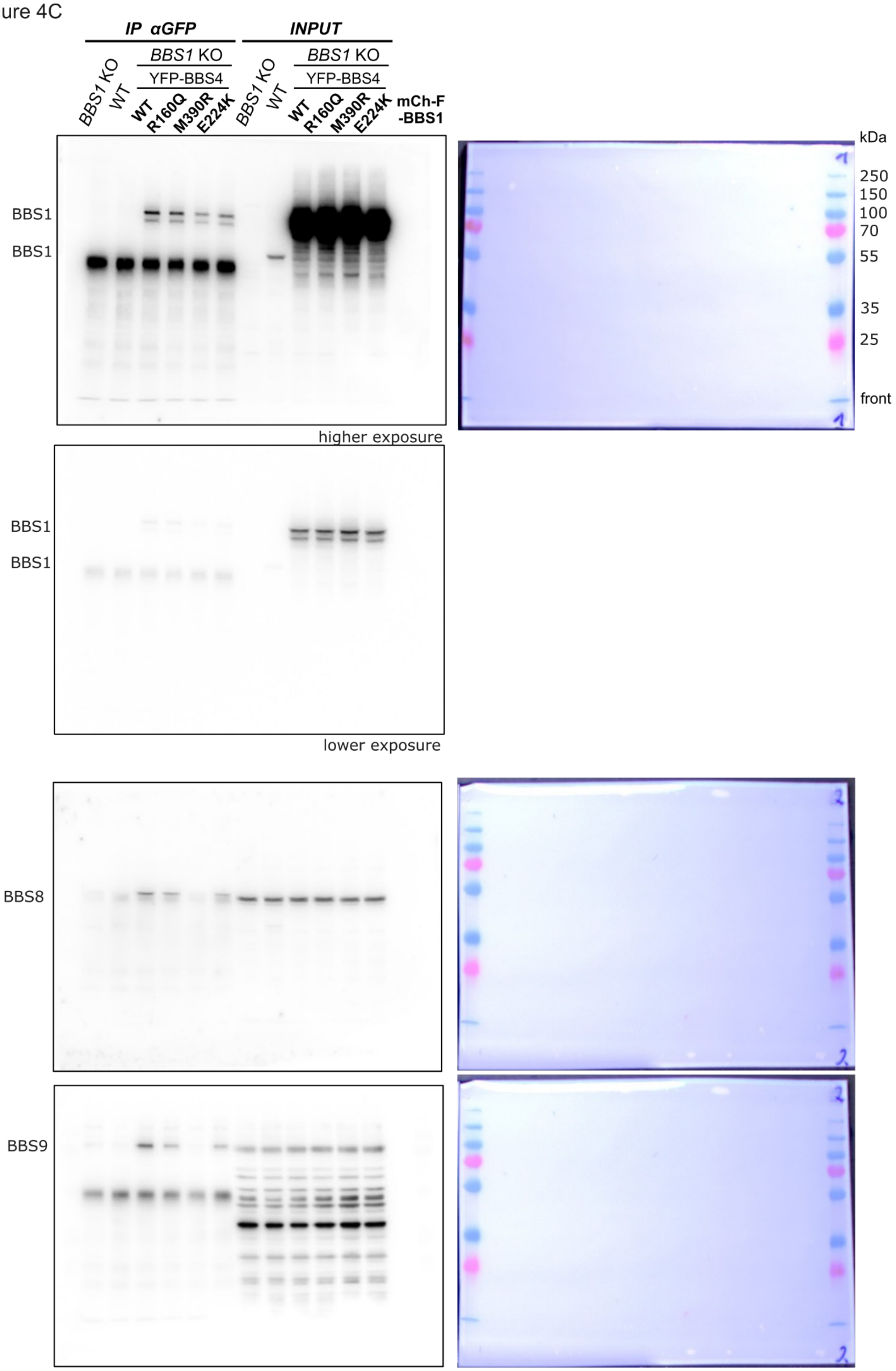
IP αGFP
INPUT
BBS1 KO
YFP-BBS4
BBS1 KO
WT
WT
R160Q
M390R
E224K
mCh-F
-BBS1
kDa
250
150
100
70
55
35
25
front
BBS1
BBS1
higher exposure
BBS1
BBS1
lower exposure
BBS8
BBS9

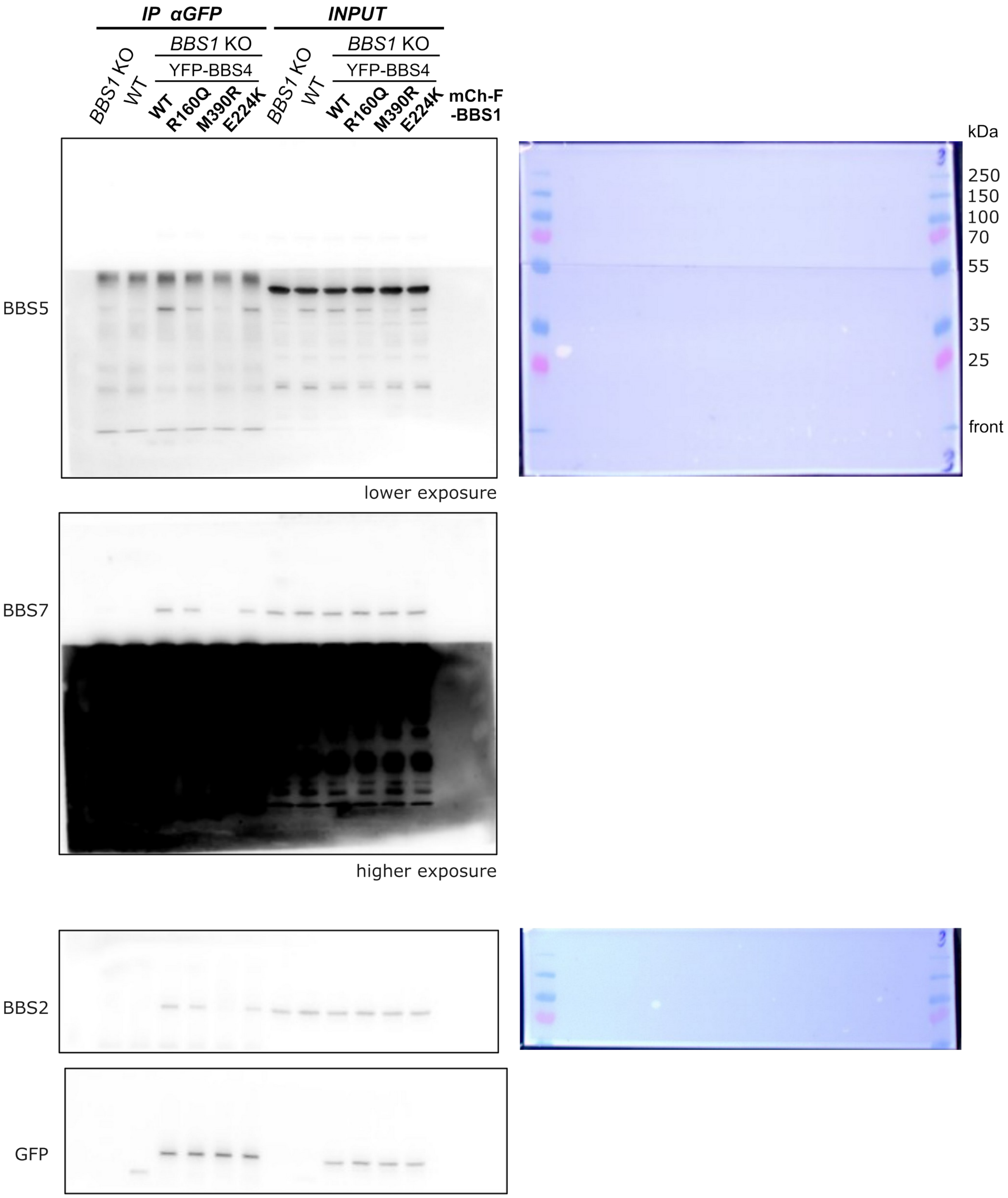
IP αGFP
INPUT
BBS1 KO
YFP-BBS4
BBS1 KO
WT
WT
R160Q
M390R
E224K
BBS1 KO
YFP-BBS4
BBS1 KO
WT
WT
R160Q
M390R
E224K
mCh-F
-BBS1
kDa
250
150
100
70
55
35
25
front
BBS5
lower exposure
BBS7
higher exposure
BBS2
GFP

Supplemental Figure 1C

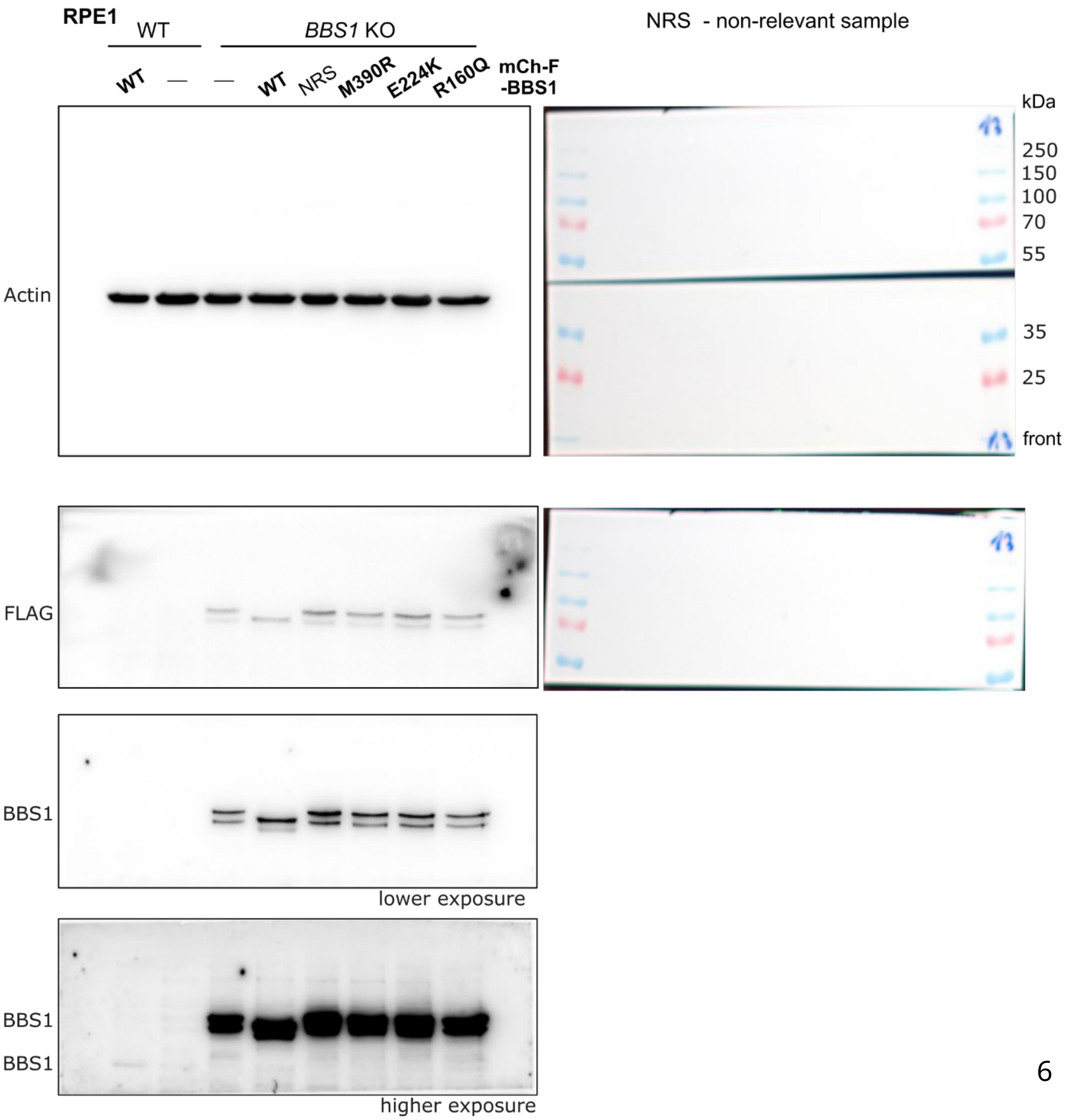

Supplemental Figure 3E

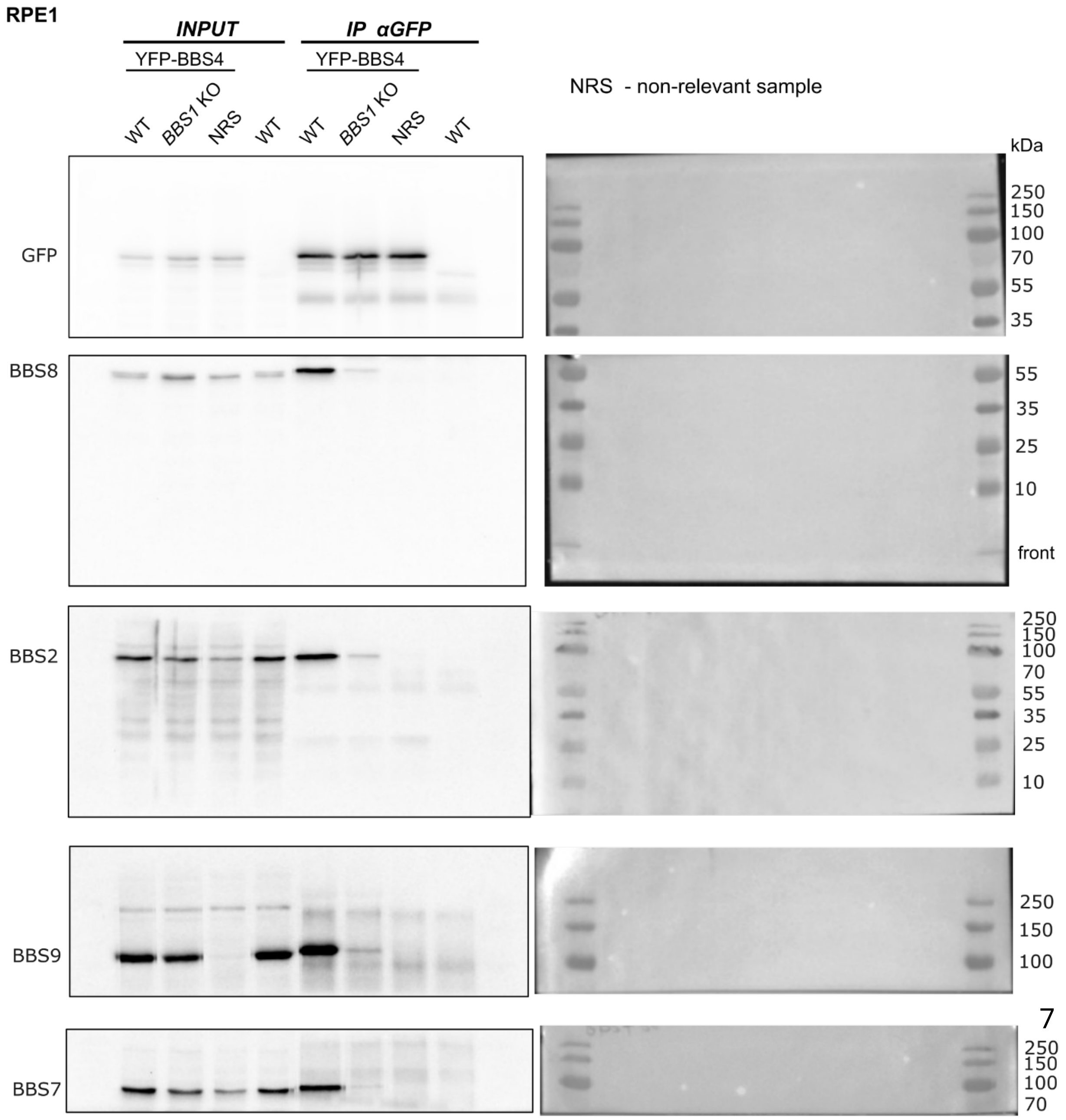

Supplement: Supplementary file 2 [file 12964_2026_3006_MOESM2_ESM.docx]
